# Global Patterns in the Evolutionary Relations Between Seed Mass and Germination Traits

**DOI:** 10.1002/ece3.71543

**Published:** 2025-06-11

**Authors:** Keyvan Maleki, Filip Vandelook, Arne Saatkamp, Kourosh Maleki, Siavash Heshmati, Elias Soltani

**Affiliations:** ^1^ Department of Horticulture and Crop Science The Ohio State University Columbus Ohio USA; ^2^ Research Department Meise Botanic Garden Meise Belgium; ^3^ Aix Marseille Université Université Avignon, CNRS, IRD, UMR IMBE Marseille France; ^4^ Department of Forestry Gorgan University of Agricultural Sciences and Natural Resources Gorgan Iran; ^5^ Department of Plant, Soil and Microbial Sciences Michigan State University East Lansing Michigan USA; ^6^ Department of Agronomy and Plant Breeding Sciences, College of Aburaihan University of Tehran Tehran Iran

**Keywords:** adaptation, climate change, germination traits, phylogeny, seed dormancy, seed mass

## Abstract

The relationship between seed mass and germination traits remains unresolved, with conflicting theoretical predictions. Some studies suggest larger seeds exhibit superior germination and post‐germination performance, while others indicate the opposite, possibly due to environmental factors and additional seed traits. To clarify this relationship, we conducted a phylogenetically informed meta‐analysis of 1889 plant species worldwide. Our findings show that seed dormancy is strongly associated with seed mass, but the direction and magnitude of this relationship vary by dormancy type, with lower seed mass in physiologically dormant seeds (−0.026) and higher seed mass in morphophysiologically (0.066) and physically dormant seeds (0.598). Trade‐offs and correlated evolution have influenced seed mass, which exhibits notable phylogenetic conservation. Contrary to predictions, larger seeds did not consistently germinate faster or to a higher percentage (−0.648 and 0.890), likely due to interactions with growth form, dormancy class, and phylogenetic history. These findings highlight the importance of considering seed traits, growth form, and evolutionary relationships when examining plant adaptation and germination strategies.

## Graphical abstract

Our research systematically reviews 1889 species to resolve conflicting theories on seed mass and germination traits. We find that the relationship is complex and varies significantly across species and dormancy types, challenging existing hypotheses and highlighting the importance of considering growth form, dormancy type, and phylogenetic relatedness in plant adaptation studies.

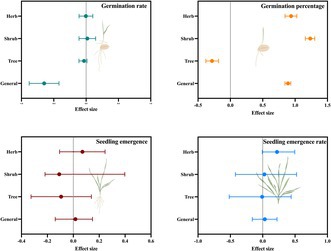

## 1 Introduction

Despite the importance of life‐history events, dormancy patterns, and the cascading effects of these adaptive traits on plant establishment and survival (Gremer, Wilcox, et al. 2020; Carta et al. 2024), our understanding of global patterns in the eco‐evolutionary correlations among adaptive traits related to germination success has received scant attention. The different roles of ecologically important life‐history traits such as seed mass are not yet fully understood. Besides seed mass, germination traits are also of considerable significance to plant evolutionary adaptations and spatial distribution (Zhang and Wang 2024). Given the pivotal role of germination and post‐germination traits in the evolutionary responses of plant species, we must attempt to determine how varying environments shape seed‐related traits by taking the correlated evolution of these traits into account.

Many aspects of a plant species' ecological strategy are determined by seed mass, either through germination or post‐germination traits or other functions, such as seed dispersal and survival (Leishman et al. 2000; Simons and Johnston 2000; Moles and Westoby 2004a, 2004b; Susko and Cavers 2008; Larios et al. 2014). Seed mass is under strong natural selection (Larios et al. 2014; Larios and Venable 2018). Studies on the implications of the variation in seed mass have illustrated various germination strategies ensuring plant survival. Seed size influences seedling competitiveness through different strategies (Carta, Fernández‐Pascual, et al. 2022; Carta, Mattana, et al. 2022; Wang et al. 2024). Larger seeds often produce more vigorous seedlings (Geritz et al. 2018), giving them a competitive advantage over later‐emerging seedlings. On the other hand, smaller seeds tend to germinate more quickly, allowing their seedlings to establish rapidly and compete effectively under transient favorable conditions (Baskin and Baskin 2014). This rapid germination response is particularly advantageous in unpredictable or regularly disturbed environments, where quick establishment may enhance survival despite limited initial resource investment (Kadereit et al. 2017; Notarnicola et al. 2023). Thus, while larger seeds are typically associated with enhanced seedling growth and competitive ability, smaller seeds may offer resilience through flexible, opportunistic germination strategies. However, these relationships remain poorly understood in the context of growth form and dormancy type, both of which significantly influence seed ecological strategies and responses to shifting climate.

When considering germination timing as an important early life‐cycle trait, characteristics related to germination, such as germination speed, must be included (Baskin and Baskin 2014; Kadereit et al. 2017; Carta et al. 2024). It is believed that germination speed, which is controlled by morphological and physiological features of seeds (Nonogaki et al. 2010), is an adaptive characteristic that evolves in response to variation in environmental conditions, climate, and life‐history strategies (Vandelook et al. 2012; Kadereit et al. 2017). Studies have shown, for example, that species with larger relative embryo sizes (embryo size in relation to the size of the whole seed) can germinate rapidly, suggesting that this might be an adaptive strategy to climatic or biotic conditions for germination and seedling establishment (Vandelook et al. 2012). Evolution has driven germination speeds to extremes in several species growing in very dry or saline habitats, resulting in germination within hours or a few days, depending on species and dormancy type (Parsons 2012; Parsons et al. 2014). While previous studies have explored the relationship between seed mass, germination speed, and internal seed morphology within the context of life‐history and functional traits (Vandelook et al. 2012; Kadereit et al. 2017; Carta et al. 2024), a comprehensive synthesis addressing the interactions among these factors at a global scale is still lacking.

Germination is directly controlled by light availability in many species (Baskin and Baskin 2014), and especially in small‐seeded ones (Milberg et al. 2000). Having small seeds is tightly linked to seed survival in the soil seed bank (Leishman et al. 2000) due to the higher likelihood of burial and poor emergence from deep soil (Xia et al. 2016). Moreover, the light requirement for germination is considered an adaptation for small‐seeded species to induce germination when seeds come closer to the soil surface and can capture gap detection signals. In contrast, large‐seeded plant species rely more often on thermal signals, such as daily temperature fluctuations, enabling them to emerge even when buried deeply (Ghersa et al. 1992; Pearson et al. 2002; Xia et al. 2016). To obtain a complete understanding of the relation between a light requirement for germination and seed mass, it is crucial to consider the impact of the interaction with seed dormancy as well, as seed dormancy may change the light requirement for seed germination (Baskin and Baskin 2014).

Seed mass and dormancy can temporally and spatially vary, depending on species and geographical origin, leading to heterogeneity in environments (Pausas et al. 2022). Seed dormancy is a strategy ensuring plant survival in an unpredictable environment by delaying germination until conditions are suitable for seedling establishment and survival or by spreading germination in time (Pausas et al. 2022; Zhang and Wang 2024). The mechanism behind variation in seed dormancy has been defined as bet‐hedging, suggesting a strategy of spreading germination over time to reduce the risk of failure by taking advantage of erratic favorable conditions (Philippi 1993; Pausas et al. 2022). The trade‐off between seed mass and dormancy is well documented (Baskin and Baskin 2014; Volis and Bohrer 2013; Chen et al. 2020). This trade‐off might be linked to survival strategies favoring some traits in a given environment to increase plant fitness. Chen et al. (2020) reported that there might have been correlated evolution between seed mass and dormancy, with the selective interaction having positive effects on geometric mean fitness. They found that small‐seeded species exhibit high levels of seed dormancy, implying that seed dormancy is an evolutionarily stable strategy of plant species in response to short spells of suitable conditions (Rubio de Casas et al. 2017; Rosbakh et al. 2023). Furthermore, Rees (1996) reported that larger seeds are likely to show lower levels of seed dormancy relative to their smaller counterparts, although Soltani et al. (2018) showed that this relationship is highly dependent on dormancy type, with seeds with physical dormancy showing the reverse pattern. These previous observations, which indicate that heavy seeds have a decreased likelihood of exhibiting dormancy, may be congruent with enhanced establishment success and reduced susceptibility to herbivory (Rees 1996). Despite these insights, the interaction between seed mass and dormancy type remains unclear, particularly with respect to the role of phylogenetic relatedness in shaping these traits across diverse ecological contexts (Soltani et al. 2018).

Given the importance of a global synthesis studying the interaction among seed mass, germination‐related traits (light requirement, germination speed and relative embryo length), and seed dormancy, this study aims to test hypotheses about the interaction between seed mass and germination and post‐germination traits, with a focus on how seed mass interacts with these traits, influences germination responses to light availability, and is mediated by phylogenetic relatedness. We used phylogenetically informed statistical methods to address the following questions: (1) Does the relationship between seed mass and germination/post‐germination traits depend on growth form and dormancy type? (2) How do seed internal morphological features, such as relative embryo size, interact with seed mass to influence the germination speed of plant species? (3) How does seed mass mediate seed germination responses to light? (4) How does phylogenetic relatedness mediate the relationship between seed mass and germination/post‐germination traits and their responses to light availability? While our study does not include biochemical parameters, we highlight the value of exploring such factors in future research to gain mechanistic insights into the physiological drivers underlying germination patterns.

## 2 Material and Methods

### 2.1 Study Selection and Data Collection

Data on seed mass and germination and post‐germination traits were collected from literature using the Web of Science database, with the objective of aggregating relevant studies covering the period from 1945 to January 2023. All the studies that fit the criteria mentioned below were used in the meta‐analysis. This process covered the following terms: seed size and germination (222), seed mass and germination (52), seed weight and germination (67), seed size and seedling emergence (43), seed mass and seedling emergence (10), and seed weight and seedling emergence (14). In total, we collected data on 1889 species. We targeted studies having data on four critical traits as follows: *germination percentage, inverse time to germination—germination rate, seedling emergence*, and *inverse time to seedling emergence—emergence rate*.

To systematically organize and report our literature search and study categorization, we adhered to the PRISMA guidelines (Figure S1; Page et al. 2021). The study inclusion requirements were as follows: (1) species or populations were tested for the variation in seed size in relation to the above‐mentioned traits, and studies reported at least two different treatments, (2) treatments included testing germination characteristics of differing seed sizes of a single species, and (3) studies with consistent seed size measures (mg. or any convertible unit except for studies reporting seed length as this unit was not convertible into mg) were taken into account. To ensure consistency in reporting seed mass across various studies, we adopted a standardized scale. We followed the methodologies outlined by Vandelook et al. (2008) and Rosbakh and Poschlod (2015) to standardize the seed mass scale for each species. Specifically, we used a milligram (mg) scale, converting any seed mass reported in grams to milligrams. Subsequently, we log‐transformed the data for all analyses. Based on these criteria, 350 studies were found that provided germination kinetics of nearly 1889 plant species. The list of all the studies used can be found in Table S1 and at https://zenodo.org/records/12682812. To obtain the suitable number of studies required for observing main effects, Rosenberg's fail‐safe numbers method was employed, which is a method estimating the average of null results that should be added to the observed outcomes to adjust the significance level (i.e., *p*‐value; data not shown) (Koricheva et al. 2013).

Plant species were classified into three groups according to their growth form: (1) trees, (2) shrubs, and (3) herbs (Moles et al. 2007). To group plants into different growth forms, we used the *Plants of the World Online database* (http://www.plantsoftheworldonline.org). To classify plant species into different dormancy types, we used the seed dormancy database compiled by Baskin and Baskin (2014) and Rosbakh et al. (2023). Using this database, we categorized plant species into one of five distinct seed dormancy types: physiological (PD), physical (PY), morphological (MD), morphophysiological (MPD), and non‐dormancy (ND). To avoid the presence of dormancy confounding with the germination traits we collected, we excluded germination tests on (partially) dormant seed lots. To achieve this, we employed the following approaches: (1) For species with known seed dormancy, we only included data on seeds that had undergone dormancy‐breaking treatments, such as stratification, after‐ripening, or other methods as described by Baskin and Baskin (2014), (2) for species lacking specific information on seed dormancy, we verified seed germination percentages and seed viability results to ensure that dormancy did not impact the findings, and (3) species that did not meet the above criteria were excluded from our analysis (see Figure S1 for details). The germination traits were categorized into two groups: seed germination traits and post‐germination traits. Seed germination traits included (1) seed germination percentage, defined as the proportion of intact, viable seeds in a population or sample that germinate (i.e., radicle emergence) in the absence of seed dormancy, and (2) seed germination rate (speed), measured as the inverse of the time required for 50% of the seeds to germinate. Post‐germination traits included (1) seedling emergence, referring to the proportion of seeds that successfully produce seedlings, and (2) seedling emergence rate, defined as the inverse of the time required for 50% of the seedlings to emerge.

We extracted information about germination responses to light from the dataset initially established by Milberg et al. (2000) encompassing 54 distinct species. We added additional data sourced from 30 scientific papers spanning the period from December 2000 to January 2023, which added 50 previously unaccounted species to the dataset. We used the Web of Science (WOS) database to extract these articles by using the keywords “seed mass and light response” and “seed mass and light requirement”. In total, we collected data on relative light germination (RLG) for 186 species.

We incorporated RLG into this study by computing relative light germination as suggested by Milberg et al. (2000):

$$\mathrm{RLG}=\mathrm{Gl}/\left(\mathrm{Gl}+\mathrm{Gd}\right)$$

where Gl = the germination percentage in light, and Gd = the germination percentage in darkness. We considered relative values preferable to germination percentages since seed batches differed in their dormancy level. RLG represents a range of values varying from 0 (germination only in darkness) to 1 (germination only in light).

Data on embryo length to seed length ratio (EL/SL) and embryo to seed surface ratio (ES/SS) were obtained mainly from a recent database on embryo length to seed length ratio and embryo (https://zenodo.org/records/5647046#.Y3X1SeSZOUl). Most data originated from Forbis et al. (2002) and were based mainly on illustrations of the internal morphology of seeds by Martin (1946). Measurements were taken from cross‐sections of mature seeds that displayed the largest area of the embryo surface. The EL/SL was calculated by dividing total embryo length by the length of the seed, measured along the longest axis from the inside of the seed coat. The ES/SS was calculated by dividing the area of the embryo by the combined area of the embryo, endosperm, and perisperm. For embryos that were curved or coiled, the embryo was divided into different segments, and the total length was calculated as the sum of the lengths of each segment. Altogether, we gathered data on EL/SL for 423 species and ES/SS ratio for 415 species (https://zenodo.org/records/12682812).

### 2.2 Statistical Approaches

#### 2.2.1 Meta‐Analysis Approach

A meta‐analysis of published data, which is a method of analyzing original data from different studies together, was performed (Koricheva et al. 2013; Borenstein et al. 2021). We used the data within studies published to synthesize a comprehensive conclusion on the eco‐evolutionary role of seed mass in controlling seed germination and post‐germination traits.

To examine whether seed mass has effects on germination and post‐germination traits, a meta‐analysis of published data was performed by running binomial phylogenetic generalized mixed models with Bayesian estimation using the Markov chain Monte Carlo (MCMC) approach (Hadfield 2010), following previous studies that used the same method for related seed traits (Fernández‐Pascual et al. 2021; Carta, Fernández‐Pascual, et al. 2022; Carta, Mattana, et al. 2022). The binomial distribution function was also used as a suitable link function for our data. This approach effectively accounts for multiple observations per species/study while considering phylogenetic relationships among different species (Carta, Fernández‐Pascual, et al. 2022; Carta, Mattana, et al. 2022; Garamszegi 2014). This approach also enabled us to account for between‐study variation owing to different scales and units used for germination traits and seed mass. In this analysis, final germination percentage, germination rate (speed), final seedling emergence percentage, and seedling emergence rate (speed) were treated as response variables while the fixed effects for our models (the predictors) included seed mass gradient (different values of seed mass reported within each study for each germination trait); see Section 2.1 for more detail on study selection and data collection. In our models, weak informative Gelman priors combined with parameter‐expanded priors for the random effects and residual variance fixed to 1 (Hadfield 2010). The model involved executing each procedure with four separate chains, each including 500,000 MCMC steps. The initial 50,000 steps of each chain were disregarded as a ‘burn‐in’ phase, and the data were taken every 100 steps (de Villemereuil and Nakagawa 2014). The outputs derived from these four chains, consisting of 500,000 MCMC, were then combined to obtain parameter estimates. The mean of these parameter outputs and their 95% credible intervals (CIs) were computed from the merged posterior distributions. To see if the parameters are significant, we examined these CIs; parameters were considered insignificant if their CIs included zero. The explanatory aspect of our analysis was evaluated through calculating the conditional *R* ^2^ (i.e., the amount of variation accounted for by both the fixed (traits) and random factors (the phylogeny)) and the marginal (i.e., the amount of variation accounted for by the fixed factors only) using the approach described by Nakagawa and Schielzeth (2013).

In this study, we addressed the potential non‐independence of data points extracted from the same articles, particularly when data were utilized across multiple experimental treatments. To account for this, we employed binomial phylogenetic generalized mixed models with Bayesian estimation using the MCMC approach. This robust statistical method allows for the inclusion of multiple observations per species or study while considering the phylogenetic relationships among species, thus controlling for non‐independence due to shared evolutionary history. Additionally, our subgroup analysis strategy, which categorizes plants into distinct classifications based on growth form and dormancy type, further mitigates the risk of conflating results from different experimental contexts.

#### 2.2.2 Multivariate Ordination Method

To test whether there is a relationship between seed mass and germination traits, principal component analysis (PCA) implemented within the R environment (FACTOMINER package) was employed (Lê et al. 2008; Rosbakh et al. 2023). We first quantified the potential relationship for each growth form through fitting univariate models. Then, predicted probabilities of the relationship for each trait to which they belong were used in the multivariate ordination.

#### 2.2.3 Binary Approach

To answer the long‐standing question of how seed mass interacts with differing types of seed dormancy, we used Bayesian logistic phylogenetically informed generalized mixed models, following Rosbakh et al. (2023). In the models, we treated seed dormancy (the response variable) as binary data. Here, 0 and 1 refer to the absence or presence of seed dormancy (0 = seed mass of non‐dormant seeds; 1 = seed mass of dormant seeds), respectively. This binary classification refers to the primary distinction in dormancy states relevant to our research question. For the fixed effects in our models, seed mass was treated as the predictor. To directly compare the response/predictor effects, we centered and scaled the variance of all the variables to be unit. To assess this relationship, the model was fitted in two distinct manners: firstly, through comparing the seed mass of non‐dormant versus dormant seeds (specifically water‐permeable versus impermeable for physical dormancy) to see how seed mass might affect the likelihood of dormancy; and secondly, by examining this correlation within each specific dormancy class separately. We aimed to understand potential variations in the seed mass‐dormancy relationship across different dormancy types, suggesting that different classes might respond differently to variations in seed mass. Consequently, every data point analyzed represented a species characterized by a particular type of seed dormancy and its associated seed mass measurement.

#### 2.2.4 Phylogenetic Regression

To determine whether there is a relationship between RLG and seed mass and between EL/SL and ES/SS ratio and germination speed, we ran a phylogenetic regression with a maximum likelihood (ML) approach. This method allowed us to calculate the phylogenetic signal and the regression model simultaneously. Pagel's *λ* was used to quantify the phylogenetic signal, as applied (Pagel 1999). When *λ* equaled zero, related taxa were not more similar than expected by chance, and the trait was evolving as in a star‐like phylogeny (Pagel 1999). In this situation, phylogenetic correction was redundant. A notable phylogenetic signal, or a grouping of trait states on the phylogenetic tree, occurred when *λ* > 0, meaning trait states of taxa were more similar than expected by chance. When the value of λ equaled 1, it indicated that the evolution of the trait is consistent with a Brownian motion model, which is characterized by a constant variance random walk. If 1 > *λ* > 0, it suggests that the trait is changing following a Brownian motion (BM) model, which assumes a consistent variance over time. Analyses were performed using the APE (Paradis et al. 2004) and nlme (Pinheiro 2011) packages in R.

More information regarding model choice and description can be found in the Supporting Information (model description section).

#### 2.2.5 Phylogenetic Tree Reconstruction

To generate a phylogeny of the studied species, we used the largest dated mega‐tree for vascular plants (Open Tree of Life) as a backbone (Jin and Qian 2019). An ultrametric phylogenetic tree containing the species in our data set was then constructed using the package *V.PhyloMaker* (Jin and Qian 2019) implemented within the R environment. *V.PhyloMaker* utilizes a mega‐tree (GBOTB.extended.tre), an updated and corrected version of the GBOTB for seed‐producing plants (Jin and Qian 2019). Additionally, we matched the taxonomic status of the plant species included in this study with The Plant List V.1.1 database (http://www.theplantlist.org/). The resulting trees are depicted with each trait mapped onto the tips using a color gradient (Figure S3).

## 3 Results

### 3.1 Relative Proportion of Seed Mass

The final database consisted of 1889 plant species representing 140 families for which we had data on germination traits and seed mass, covering all three growth forms included (Figure S2). The species included in this study had a seed mass range varying from 0.0001 to 10,000 mg. Seeds of herbs mostly occurred within a range varying from 0.01 to 100 mg, with more than 80% of species concentrated in the 0.1–100 mg range (Figure S4a,c). Most tree species tend to have a seed mass between 0.1 and 10,000 mg, with an average value of 488.4 ± 403 mg (Figure S4a). The number of tree species is normally distributed across the seed mass gradient, with more than 15% of species showing heavy seeds. Shrubs are mostly concentrated in the 0.01–10 mg range of seed masses, which is very similar to the trend reflected in herbs.

Seed mass ranges varied also among seed dormancy types, with some groups like PD and ND having a higher frequency in the lower seed mass categories (Figure S4b,d). MD, MPD, and PY showed heavier seeds relative to PD and ND, with more than 50% of these species having a higher frequency in the higher seed mass categories (Figure S4b,d).

### 3.2 The Relationship Between Seed Mass and Germination Traits

**FIGURE 1:**
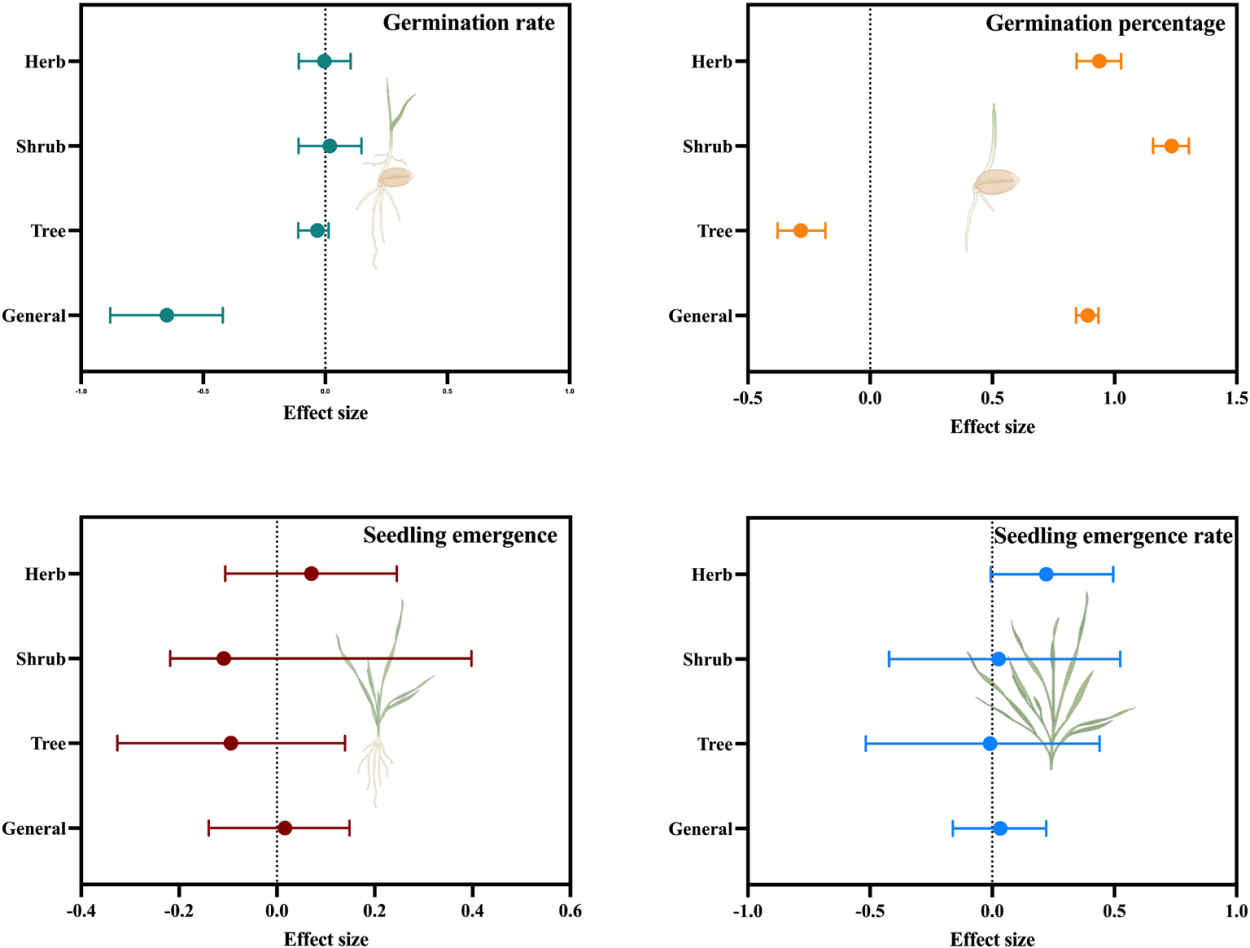
Seed mass effects on germination traits of three distinct growth forms according to the binomial phylogenetic mixed models with Bayesian estimation (MCMCglmms). Closed symbols represent the posterior means of the interaction effect size. Horizontal bars indicate the 95% credible intervals. Dashed vertical line denotes zero effect. In the top panel, germination rate and germination refer to speed of germination and percentage of germination, respectively. In the bottom panel, seedling emergence and seedling emergence rate indicate seedling emergence percentage and speed, respectively.

The univariate models predicting the distribution of seed mass and germination traits revealed that germination traits (germination percentage, germination rate) show contrasting responses when taking growth form into account. A significantly negative effect of seed mass on germination percentage was observed for trees (posterior mean: −0.284), suggesting that small tree seeds germinate to the highest percentage (Figure 1; Table 1). Herbs and shrubs showed a significant positive relation between seed mass and germination percentage (posterior mean: 0.938 and 1.234, respectively). The general trend of seed germination percentage (pooled data) was also positively related to seed mass (posterior mean: 0.890). We found no significant effect of seed mass on germination rate for growth forms separately. When pooling data together (general trend; posterior mean: −0.648), germination rate was significantly negatively correlated with seed mass (Figure 1; Table 1).

**TABLE 1:** Effects of seed mass on germination traits across different growth forms using a global database as estimated by Bayesian logistic phylogenetically informed generalized mixed models.

Seed mass						
Germination traits	Mean parameter estimate					
	Trees	Shrubs	Herbs	General (pooled data)	*R* ^2^ marginal	*R* ^2^ conditional
Germination	**−0.284**	**1.234**	**0.938**	**0.890**	0.02	0.80
Germination rate	−0.031	0.019	−0.004	**−0.648**	0.04	0.87
Seedling emergence	−0.094	−0.108	0.070	0.0165	0.06	0.81
Seedling emergence rate	−0.009	0.026	**0.221**	0.032	0.09	0.88

*Note:* Bold values refer to estimates significantly different from zero (*p* < 0.001).

### 3.3 The Relationship Between Seed Mass and Post‐Germination Traits

No significant relation between seed mass and seedling emergence was detected, neither for the pooled data (0.0165) nor for individual life forms (Figure 1; Table 1). The seedling emergence rate was significantly positively related to seed mass for the pooled data (posterior mean: 0.032) and for herbs (posterior mean: 0.221) in particular, although this relationship was only significant for herbs.

### 3.4 The Relation Between Seed Dormancy Type and Seed Mass

**FIGURE 2:**
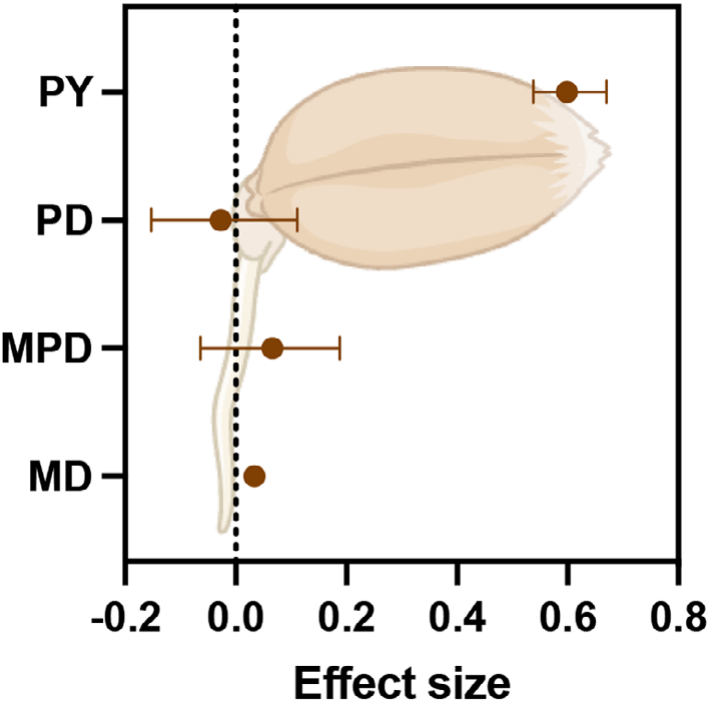
Seed mass effects on dormancy levels for four dormancy types according to the binary approach. Closed circles show the posterior means of the interaction effect size. Horizontal bars represent the 95% credible intervals. Dashed vertical line denotes zero effect. MD‐morphological dormancy; MPD‐morphophysiological dormancy; PD‐physiological dormancy; PY‐physical dormancy.

The relationship between seed dormancy and seed mass was highly dependent on dormancy type, with MD (posterior mean: 0.034), MPD (posterior mean: 0.066) and PY (posterior mean: 0.598) being significantly positively related to seed mass, suggesting that seeds of species with MD, MPD, and PY are heavier (Figure 2; Table 2). A negative relation with seed mass was observed for species with PD (posterior mean: −0.026).

**TABLE 2:** Effects of seed mass on dormancy levels across different seed dormancy types as estimated by Bayesian logistic phylogenetically informed generalized mixed models.

Seed mass			
Dormancy class	Mean parameter estimate	*R* ^2^ marginal	*R* ^2^ conditional
PD	−0.026	0.03	0.85
PY	**0.598**	0.02	0.83
MD	**0.034**	0.07	0.83
MPD	**0.066**	0.05	0.82

*Note:* Bold values refer to estimates significantly different from zero (*P* < 0.001).

### 3.5 Correlation Between Seed Mass and Light Response (RLG)

**FIGURE 3:**
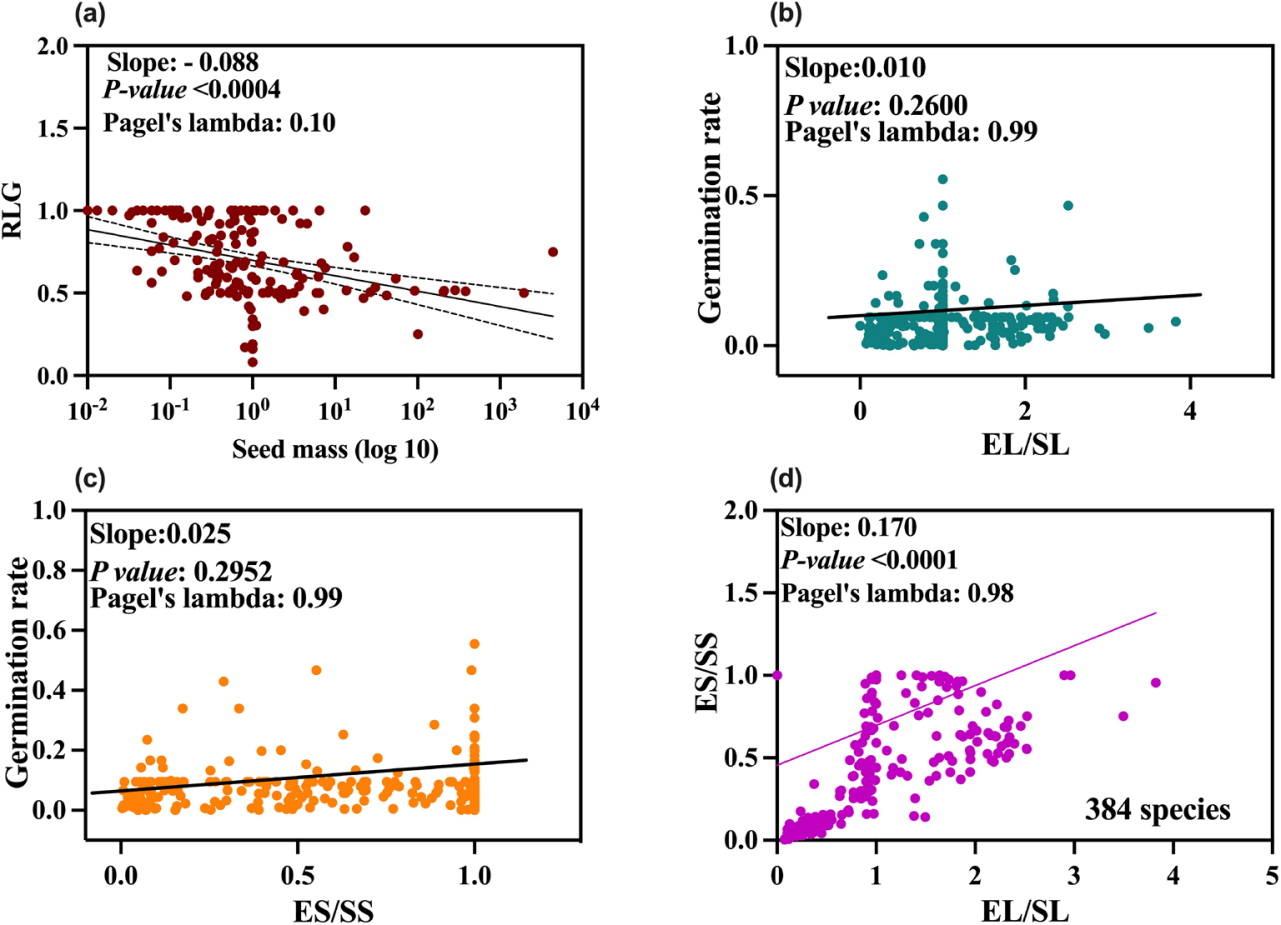
(a) Correspondence between seed mass and relative light germination (RLG). Phylogenetic regression of RLG on seed mass. (b) Phylogenetic regression of germination rate on EL/SL (embryo length/seed length). (c) Phylogenetic regression of germination rate on ES/SS (embryo size/seed size). (d) Phylogenetic regression of EL/SL on ES/SS. Fitted lines were estimated via phylogenetic regression approach (Revell 2010).

Light response was significantly negatively correlated with seed mass (Figure 3a). Small‐sized seeds thus showed higher germination in light (slope: 0.088; *p* value: 0.0004).

Notably, a parallel pattern emerged within the spectrum of plant life forms (Figure S5), reflecting the previously observed trend across all species encompassed in the analysis (Figure 3a). This indicates that the light responsiveness of forbs (slope: −0.137) displays a relatively diminished reliance on light intensity compared to the responses of other life forms, including shrubs (slope: −0.027) and annual grasses (slope: −0.059). Moreover, the phylogenetic signal λ observed for RLG in different growth forms was close to 0.

### 3.6 Relationship Between Germination Rate and EL/SL and ES/SS

A positive but non‐significant (*p* < 0.096) correlation was found between germination rate and EL/SL, with a phylogenetic regression slope of 0.042 (Figure 3b). A similar trend was also reflected in the relationship between germination rate and ES/SS, with a phylogenetic regression slope of 0.046, showing a smooth but non‐significant correlation between germination rate and ES/SS (Figure 3c; *p* = 0.244). Interestingly, phylogenetic regression showed that both EL/SL and ES/SS are phylogenetically conserved (Pagel's λ: 0.99; Figure 3).

### 3.7 Phylogenetic Relatedness

Our models exhibited comparatively weak explanatory power, as the marginal R^2^ was rather limited in all the models. The cumulative amount of variance accounted for by our models was highest in seedling emergence rate (*R* ^2^ = 0.09), followed by seedling emergence (*R* ^2^ = 0.06) and germination rate (*R* ^2^ = 0.04). The cumulative amount of variance accounted for by our models was lowest in germination percentage (*R* ^2^ = 0.02). Interestingly, the conditional *R* ^2^, the major contributor to phylogenetic relatedness, was higher in all models.

### 3.8 Multivariate Ordination

**FIGURE 4:**
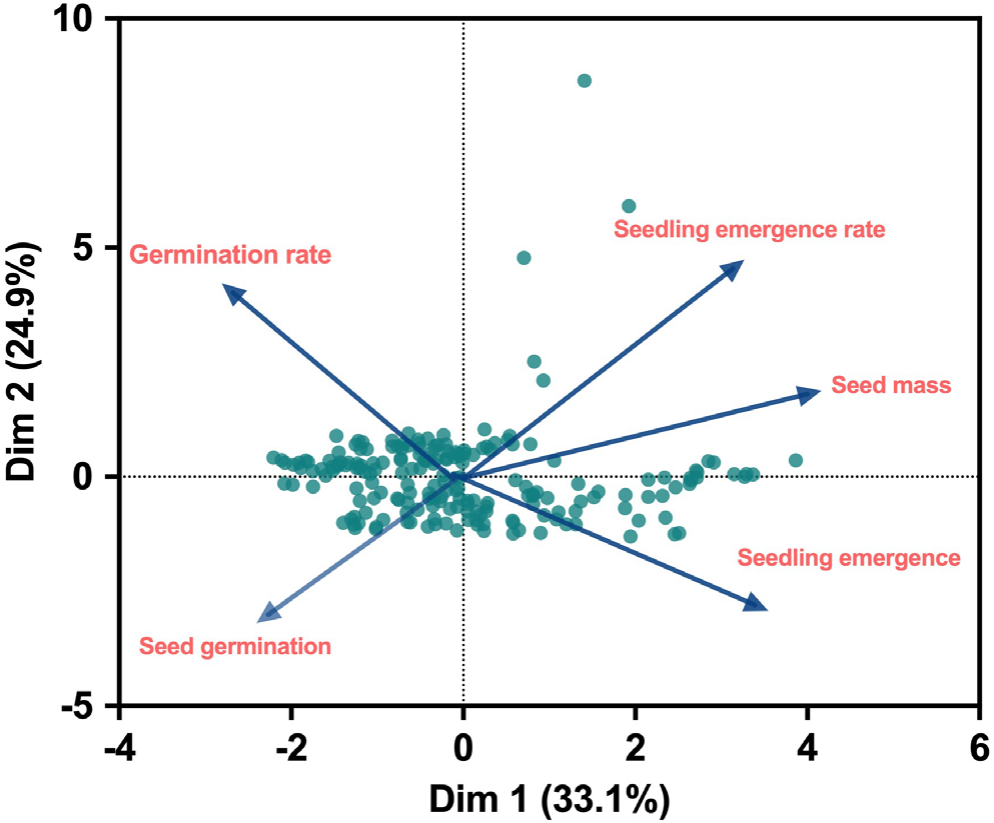
Principal component analysis (PCA) ordination representing the relationships among germination traits and seed mass. Each closed point refers to a species.

Two principal components summarized the variation in seed mass, germination traits, and growth form by taking 58% of the total variation into account (Figure 4). 33% of the variance was explained by the first component (PC1). PC1 was mainly loaded on seedling emergence, seed mass, and seedling emergence rate (Figure 4). Thus, the first principal component separates species with heavier seed mass and higher seedling emergence rate and percentage. 24.9% of the total variation observed was explained by the second component (PC2). Germination rate and germination percentage were the main contributing variables on axis 2, which are classified mostly into trees and herbs (Figure 4).

## 4 Discussion

The relationship between germination traits and seed mass varied considerably among growth forms. Trees showed a consistent negative relationship between seed mass and germination and seedling emergence rate. Our findings highlight that responses of germination traits to seed mass gradients differ between growth forms. Seed mass variation also depended on seed dormancy class. Seeds with PD were negatively correlated with seed mass, while seeds with PY, MD, and MPD showed a positive relationship with seed mass.

### 4.1 Correlation Between Seed Mass and Germination Traits

According to theory, it was believed that seed mass interacted with germination, with heavier seeds germinating to the highest percentage (Venable and Brown 1988). Our results suggest that germination traits are affected by the seed mass gradient, but this relationship is highly dependent on growth form. We found that germination is positively correlated with seed mass in herbs and shrubs, but a negative relationship was observed for trees. The observed negative relationship between seed mass and germination percentage in trees may reflect adaptations specific to forest ecosystems, where smaller seeds often germinate more readily due to their ability to disperse into the understory and establish in gaps where light availability is limited (Dalling et al. 1998; Moles and Westoby 2004a, 2004b; Umaña et al. 2020; Wang et al. 2024). Small‐seeded trees, as opposed to large‐seeded species, may capitalize on transient gaps in the forest canopy, allowing rapid establishment and maximizing recruitment opportunities when conditions are momentarily favorable (Augspurger 1984). Additionally, the high germination rate of small‐seeded forest species may be an evolutionary response to competitive pressures in densely vegetated environments, where prompt establishment following seed dispersal enhances survival prospects (Connell and Green 2000; Maleki, Soltani, Chmielarz, et al. 2024). However, this pattern may not hold for shrubs and herbaceous plants, which often grow in more open or patchy habitats where competition for light is less limiting. In such environments, factors like soil moisture, temperature fluctuations, or disturbance regimes (e.g., grazing, fire) may exert stronger selective pressures on germination strategies, resulting in a weaker or different relationship between seed mass and germination percentage. Furthermore, many shrub and herb species rely on persistent soil seed banks, which can decouple the immediate germination response from seed mass, as smaller seeds are more likely to persist in the seed bank and germinate over time. These patterns highlight a critical ecological strategy in forest regeneration, as seed mass and germination patterns often correlate with light‐demanding traits and dispersal capabilities (Grubb 1998). Furthermore, this may be explained by the potential competition from neighboring and maternal plants that exist in the forest understory, which in turn may select for long‐distance dispersal or increased levels of seed dormancy, requiring smaller and lighter seeds to easily disperse or to enter deeper soil layers to form a soil seed bank. Additionally, most tree species exhibit PD (Rosbakh et al. 2023), which favors delayed germination and requires dormancy‐breaking agents (i.e., stratification and after‐ripening) to overcome dormancy, leading to cueing of germination to periods with suitable conditions (Escobar et al. 2021; Maleki et al. 2022; Just et al. 2023).

Seeds are able to germinate at various speeds. The time required to shift from the beginning of imbibition to radicle protrusion may vary depending on the seed coat thickness and environmental factors underlying germination (Baskin and Baskin 2014). Contradictory results have been reported regarding the relationship between seed mass and germination rate; in some of them, germination rate is significantly and negatively correlated with seed mass (Norden et al. 2009) while in other reports, a temperature‐dependent positive and significant relationship was found between seed size and germination rate (Vandelook et al. 2012; Steiner et al. 2019; Carta, Mattana, et al. 2022).

Theoretical studies emphasized that larger seeds may germinate faster, and this argument is contingent on the survivorship of large‐sized seeds due to higher post‐dispersal predation (Tangney et al. 2020), indicating that a high pressure imposed by post‐dispersal predation might promote rapid germination in larger seeds (Janzen 1971; Louda 1989; Tangney et al. 2020; Pausas and Lamont 2022). The preceding discussion may also explain the positive correlation between seed mass and germination rate observed for shrubs. Furthermore, a study elaborating on data for hundreds of plant species with worldwide distribution suggested that there is no evidence of a negative relationship between seed mass and seed survivorship (Moles and Westoby 2003). We found that germination rate indeed accelerates with increasing seed mass in shrubs, which highlights the complexity of the correlation between seed mass and germination rate. This observation aligns with theoretical predictions and supports the notion that seed mass and germination rate have coevolved as an alternative mechanism to cope with unpredictable environments (Venable and Brown 1988; Kadereit et al. 2017).

### 4.2 Correlation Between Seed Mass and Post‐Germination Traits

Much attention has been given to the correlation between seed mass and seedling emergence (Singh and Saxena 2009). Studies have shown that seedling emergence is associated with germination rate and timing (Mishra et al. 2014; Cao et al. 2018). The seedling emergence rate may not be sensitive to seed dormancy because it is not just a function of the germination process but is also influenced by other factors, such as root growth, shoot growth, or resource availability (Gardarin et al. 2016). Furthermore, the rate of seedling emergence could be more related to the underlying developmental processes, such as the timing of organ initiation or tissue differentiation, rather than seed dormancy status (Vandelook and Van Assche 2008).

Our results showed that, except for trees, seedling emergence rate increases as the seed mass increases, and this relationship was statistically positive when fitting the model on pooled data. An explanation for the fact that seedling emergence rate of larger seeds is faster than small ones is that large‐seeded species are prone to higher post‐dispersal seed predation and herbivory, meaning that faster emergence of larger seeds favors survival through establishment prior to being destroyed or grazed by herbivores (Dalling et al. 2011; Cao et al. 2018). More interestingly, studies have reported that the reason given for higher seedling emergence rates of larger seeds is that seedlings grown from large seeds have higher amounts of inherent carbon metabolic rates as well as seed resource consumption rates, giving rise to more vigorous seedlings (Green and Juniper 2004, Quero et al. 2007). However, other studies arrived at a different conclusion (Norden et al. 2009). For example, no relationship between seed mass and seedling survival was reported (Leishman 1999). These inconsistencies suggest that the relationship between seed mass and seedling emergence may be context‐dependent, influenced by factors such as species traits, environmental conditions, seed dormancy, and growth forms. Also, in our study, less data was available on seedling emergence and seedling emergence rate within scientific literature, which calls for further research on these ecologically meaningful traits.

### 4.3 Relation Between Seed Mass and Growth Forms and Dormancy Class

Classifying seed mass into life form classes and plotting frequency histograms of seed mass distributions revealed that the magnitude of variation in seed mass varies among species and life forms, a pattern also observed by Carta, Fernández‐Pascual, et al. (2022) and Carta, Mattana, et al. (2022). It has been well documented that the latitudinal gradient explains much of the global variation (> 51%) in seed mass (Moles et al. 2007), but this is the first report of the magnitude of seed mass variation within different dormancy classes. Previous studies have shown that global variation in seed mass might be an important driver of species distribution, with more than 46% of variation in species distribution being accounted for by seed mass, seed mass variability, and seed dispersal mode (Moles et al. 2007; Chen et al. 2022). Variation in seed mass can explain geographical ranges within which plants can thrive, leading to the different ecological strategies that have evolved in plants (Chen et al. 2022).

It has been shown that the level of dormancy varies across species (Baskin and Baskin 2014) and even among different populations of the same species (Maleki et al. 2022), resulting in divergent requirements for dormancy loss in seeds. Our results show that the magnitude of the increase in variation of seed mass was related to both dormancy class and growth forms, which is consistent with previous studies exploring the seed mass gradient in different growth forms (Moles and Westoby 2003; Moles et al. 2007). Moreover, empirical studies have reported that there is a trade‐off between seed mass and seed dormancy (e.g., Chen et al. 2020). This relationship between seed mass and dormancy can result from the trade‐offs between seed mass and dormancy incurred by morphological constraints and the allocation cost of making larger seeds (Smith and Fretwell 1974). Moreover, the preceding studies implied that selection might have operated on these two traits, either by increased dispersal or increased dormancy, meaning that a relationship between dormancy and seed mass cannot be excluded (Philippi 1989; Volis and Bohrer 2013). The fact that seed mass has been under strong natural selection is well known (Larios et al. 2014; Larios and Venable 2018), which in turn may prove that some environmental conditions may favor specific strategies, which can be either heavier seeds or increased dormancy level (Rees 1996).

### 4.4 Eco‐Evolutionary Relationship Between Seed Mass and Dormancy

Unlike theoretical evidence suggesting that seed mass is independent of seed dormancy (Volis and Bohrer 2013), we found that a relationship exists between seed mass and seed dormancy, although this relationship is complex and varies among dormancy classes. Our results showed that the relationship between seed mass and dormancy highly depends on dormancy classes, with PD being negatively correlated with seed mass, while PY, MD, and MPD are positively correlated with seed mass. A higher seed mass in seeds with MD and MPD is probably related to the fact that these species show post‐dispersal embryo growth, where the embryo grows inside the seed after dispersal but before germination. Species with MD and MPD typically grow in shaded environments such as forests because of the predictable and humid conditions required for embryo growth. Species growing in forests also typically have larger seeds (Moles et al. 2005a, 2005b), which explains the positive relation with MD and MPD (Baskin and Baskin 2014; Vandelook et al. 2012).

The higher seed mass we observed in seeds with physical dormancy is in agreement with the crypsis hypothesis invoked by Paulsen et al. (2013) to explain the evolution of the water‐impermeable seed coat as a predation avoidance mechanism. In previous studies, it was already observed that physical dormancy is absent from lineages with predominantly small seeds (Leishman et al. 1995; Leishman and Westoby 1998; Šerá and Šerý 2004). In contrast, in a study on seeds of 14 species with physical dormancy, it was shown that the threshold temperature for seed dormancy breaking is higher in small seeds than in larger seeds (Liyanage and Ooi 2018). From a plant strategy perspective, this might be a mechanism for smaller seeds that respond to fire as a germination cue to avoid germination too deep in the soil, as the temperatures required to overcome physical dormancy may not be reached when seeds are buried too deep in the soil (Liyanage and Ooi 2018). In a meta‐analysis of the effects of frugivory (endozoochory) on seed germination, results indicated that the germination percentage of all seed mass categories, in seeds with physical dormancy, was increased by gut passage, and the magnitude of the increase in germination percentage was higher in large seeds than in medium and small seeds (Soltani et al. 2018). This suggests that the water‐impermeable seed coat provides better protection in adverse environments, enabling seeds to reach larger sizes. On the other hand, once the seed coat becomes permeable, the protective function is lost, and the large seeds profit from germinating rapidly (Dalling et al. 2011).

### 4.5 Correlated Evolution of the Germination Response to Light and Seed Mass

Light requirements for germination have been studied for decades (Milberg et al. 2000; Baskin and Baskin 2014; Xia et al. 2016). Smaller seeds more often require light to complete germination, while large‐seeded species seem less dependent on light (Grime et al. 1981; Milberg et al. 2000). The reason given for this is that large‐seeded species can emerge successfully from deeper layers of soil than light can penetrate (Del Arco et al. 1995). In addition, light serves as a gap detection mechanism, which benefits small‐seeded pioneer species that establish after a disturbance in the vegetation (Vandelook et al. 2008). Our results indicate that as seed mass increases, seeds become less dependent on light to complete germination. These findings are consistent with the hypothesis proposed by Milberg et al. (2000) and the empirical framework of Grime et al. (1981), indicating that light as an environmental signal triggering germination becomes less important in species with relatively large seeds. However, it is worth noting that RLG differs considerably and that a high amount of the variation in RLG is explained by seed mass. Furthermore, many species germinated better in light than in darkness (points below the regression line in Figure 3). Since the slope is negative, it is assumed that light consistently seems to be less important in large‐seeded species. In agreement with our findings, studies have shown that there is a trade‐off between seed mass and light responses (Milberg et al. 2000; Rubio de Casas et al. 2017; Santana et al. 2020).

### 4.6 The Correlation Between EL/SL and ES/SS and Germination Rate

A positive relation between relative embryo size (expressed as EL/SL or ES/SS) and germination speed has been observed in both Apiaceae (Vandelook et al. 2012) and Amaranthaceae (Vandelook, Newton, et al. 2021). This relation has been explained by the fact that the transfer of nutrients from endosperm or perisperm to the growing seedling is time‐consuming. In our global study, we have shown that the global relationship between germination speed and relative embryo size is non‐significant. This agrees with the results of Verdu (2006), who equally showed that there was no relationship between relative embryo size and germination speed using a global data set. The reason for this discrepancy may be that different plant families exhibit widely different dormancy mechanisms and seed morphologies that may mask potential relationships between relative embryo size and germination speed.

## 5 Conclusion

Germination is a critical stage in the plant life cycle, where seed mass plays a pivotal role in shaping early life‐history strategies. Our findings indicate that the relationship between seed mass and germination traits is complex and influenced by factors such as growth form, dormancy type, and environmental conditions. While seed mass often correlates with germination success in herbs and shrubs, the pattern reverses in trees, where smaller seeds may confer advantages for rapid establishment in light‐limited forest understories. Furthermore, seed dormancy, particularly physical dormancy, is intricately linked to seed mass, with larger seeds often exhibiting strategies that delay germination to synchronize with optimal environmental conditions.

The co‐evolution of seed mass and dormancy highlights the diverse ecological strategies plants employ to enhance survival in fluctuating environments. The variations observed in light sensitivity during germination and the correlated evolution of seed mass and dormancy traits underscore the adaptive significance of seed mass in plant ecology. Future research should continue exploring these mechanisms to deepen our understanding of the evolutionary dynamics driving seed traits and their broader ecological implications. Comprehensive studies that integrate genetic, biochemical, and ecological data will be essential in unraveling the complex interplay between seed mass, dormancy, and environmental adaptation.

## Author Contributions

**Keyvan Maleki:** conceptualization (equal), data curation (equal), formal analysis (equal), investigation (equal), methodology (equal), resources (equal), software (equal), validation (equal), visualization (equal), writing – original draft (equal), writing – review and editing (equal). **Filip Vandelook:** data curation (equal), formal analysis (equal), investigation (equal), methodology (equal), supervision (equal), writing – review and editing (equal). **Arne Saatkamp:** formal analysis (equal), investigation (equal), methodology (equal), supervision (equal), writing – review and editing (equal). **Kourosh Maleki:** data curation (equal), formal analysis (equal), investigation (equal), methodology (equal). **Siavash Heshmati:** data curation (equal), writing – review and editing (equal). **Elias Soltani:** conceptualization (equal), formal analysis (equal), project administration (lead), supervision (equal), validation (equal), visualization (supporting), writing – original draft (equal), writing – review and editing (equal).

## Conflicts of Interest

The authors declare no conflicts of interest.

## Supporting information


Data S1

## Data Availability

All data and codes used in the current study can be found at https://zenodo.org/records/12682812.
