# Supplementary material for: Global Patterns in the Evolutionary Relations Between Seed Mass and Germination Traits
Source: Ecol Evol. 2025 Jun 11;15(6):e71543. doi: 10.1002/ece3.71543 (PMC12158795; doi:10.1002/ece3.71543)
Supplement: Supplementary file 1 — Data S1 [file ECE3-15-e71543-s001.docx]

**Global patterns in the evolutionary relations between seed mass and germination traits**

**Supplementary Model Description**

In the current study, we employed a suite of statistical models to investigate the eco-evolutionary role of seed mass in controlling seed germination traits. These models were chosen for their ability to integrate phylogenetic information and account for the non-independence of species related by common ancestry.

***Binomial phylogenetic generalized mixed models (PGLMM) with Bayesian estimation via the Markov chain Monte Carlo (MCMC)***

We employed binomial phylogenetic generalized mixed models (PGLMM) with Bayesian estimation via the Markov chain Monte Carlo (MCMC) approach. This method is particularly advantageous for its ability to integrate phylogenetic relationships, thereby addressing non-independence due to shared ancestry—a critical consideration in evolutionary biology (Hadfield & Nakagawa, 2010; Garamszegi, 2014). Additionally, it accommodates variations in measurement scales and units across studies, ensuring a harmonized and robust analysis of seed mass and germination traits.

The MCMC method excels in estimating complex posterior distributions that are otherwise challenging to compute directly. It is especially suited for PGLMM due to its proficiency in managing the intricacies of hierarchical data structures and its comprehensive integration of random effects, encapsulating the inherent uncertainty in model parameters (Van Ravenzwaaij et al., 2016; Bhattacharya et al., 2016; Speagle, 2019). By incorporating random effects, the model can account for between-study variation and the phylogenetic signal, enhancing the reliability and validity of our results (Hadfield, 2010).

A pivotal component of the MCMC process is the 'burn-in' period, during which the algorithm reaches a state of equilibrium, thus mitigating potential biases introduced by initial arbitrary parameter values. This phase ensures that subsequent samples are representative of the target distribution, thereby bolstering the reliability of the parameter estimates derived from the model (Gilks et al., 1996; Gelman et al., 2013). The use of weakly informative priors in combination with parameter-expanded priors for random effects further enhances the model's robustness, preventing overfitting and ensuring the convergence of the MCMC chains (Gelman et al., 2008; Carpenter et al., 2017).

In our analysis, we treated final germination percentage, germination rate, final seedling emergence percentage, and seedling emergence rate as response variables, with seed mass gradients serving as predictors. By executing multiple MCMC chains and combining their outputs, we obtained reliable parameter estimates and their 95% credible intervals, ensuring the statistical rigor and ecological relevance of our findings (Hadfield & Nakagawa, 2010; Fernández-Pascual et al., 2021).

***Multivariate Ordination Method***

The Multivariate Ordination Method, specifically Principal Component Analysis (PCA), employed in our study serves as a sophisticated tool for discerning patterns and relationships within multivariate data sets. This method is particularly beneficial for its ability to reduce the dimensionality of complex data while preserving the most significant variance among variables, thereby facilitating a clearer understanding of the underlying structures. The application of PCA in phylogenetic analysis is well-documented and validated, with numerous studies underscoring its utility in ecological and evolutionary research (Jombart et al., 2010; Collyer & Adams, 2018).

PCA simplifies the complexity of multivariate data, making it easier to visualize and interpret. It helps in identifying patterns that may not be immediately apparent in high-dimensional data, effectively highlighting key axes of variation (Ringnér, 2008). By incorporating phylogenetic information, PCA aligns phenotypic data with evolutionary trends, providing insights into macroevolutionary patterns and enabling researchers to understand how traits have evolved over time (Revell, 2009; Collyer & Adams, 2021).

In the context of our study, PCA was instrumental in testing the relationship between seed mass and germination traits across different growth forms. By fitting univariate models and using the predicted probabilities in the PCA, we effectively quantified and visualized the potential relationships for each trait. This approach offered a nuanced view of how these traits interact with seed mass, shedding light on the eco-evolutionary dynamics at play. The visual outputs of PCA, such as biplots, allowed us to discern clusters and trends that are critical for interpreting the data in an evolutionary framework (Wold et al., 1987; Jolliffe & Cadima, 2016).

***The Binary Approach***

The Binary Approach in our study is implemented through Bayesian logistic phylogenetically informed generalized mixed models. This method is particularly suited for examining traits that are naturally dichotomous, such as the presence or absence of seed dormancy. By coding seed dormancy as a binary variable (0 for non-dormant seeds and 1 for dormant seeds), we can directly assess the influence of seed mass on dormancy states across different species.

This method allows for a straightforward evaluation of the relationship between a binary trait and its predictors while incorporating evolutionary history. It acknowledges that traits do not evolve in isolation but are influenced by the phylogenetic lineage of the species (Ives & Garland, 2010). This phylogenetic consideration is crucial as it accounts for the shared evolutionary history among species, reducing the risk of Type I errors and providing more accurate inferences about trait evolution (Felsenstein, 1985).

By comparing seed mass across dormancy states, we gain insights into how seed size may predispose seeds to certain dormancy types. This methodological choice is validated by its successful application in previous studies where binary traits were analyzed within a phylogenetic framework to understand evolutionary patterns and processes (Ives & Garland, 2010; Rosbakh et al., 2023). These studies have demonstrated the effectiveness of this approach in revealing the evolutionary dynamics underlying trait distributions.

Moreover, the Bayesian framework enhances the model's flexibility and robustness. It allows for the incorporation of prior knowledge and the generation of credible intervals for parameter estimates, which provide a more nuanced understanding of the uncertainty associated with these estimates (Gelman et al., 2013). The use of logistic regression within this framework facilitates the interpretation of odds ratios, offering clear insights into the strength and direction of associations between seed mass and dormancy states (Hosmer et al., 2013). In our study, the Binary Approach provides a robust tool for dissecting the complex dynamics between seed mass and dormancy. By analyzing how seed mass influences dormancy across a phylogenetic context, we contribute to a comprehensive understanding of seed trait evolution and the adaptive significance of seed dormancy strategies in plants.

***Phylogenetic Regression with Maximum Likelihood (ML) Approach***

Lastly, we applied phylogenetic regression with a maximum likelihood (ML) approach to determine the relationship between relative light germination (RLG) and seed mass, as well as the embryo length to seed length (EL/SL) and embryo surface to seed surface (ES/SS) ratios and germination speed. This method is pivotal in evolutionary biology as it integrates phylogenetic information into regression models, accounting for the shared evolutionary history among species.

Pagel’s λ was used to quantify the phylogenetic signal, which measures the extent to which a trait's variance is influenced by phylogeny (Pagel, 1999). Analyses were performed using the APE and nlme packages in R (Paradis et al., 2004; Pinheiro et al., 2019). Pagel's λ ranges from 0 to 1, where λ = 0 indicates no phylogenetic signal, suggesting that the traits evolve independently of phylogeny, and λ = 1 indicates a strong phylogenetic signal, consistent with a Brownian motion model of evolution. Values between 0 and 1 suggest varying degrees of phylogenetic influence.

The maximum likelihood approach allows for the simultaneous calculation of the phylogenetic signal and the regression model, providing a nuanced understanding of trait evolution. This dual estimation is essential for accurately interpreting the evolutionary dynamics of traits, as it accounts for both the trait's evolutionary history and its relationship with other traits (Revell, 2010).

In our study, phylogenetic regression was instrumental in exploring how RLG, EL/SL, and ES/SS ratios correlate with seed mass and germination speed across species. This method not only helps in identifying significant evolutionary patterns but also in understanding the ecological and functional implications of these traits (Freckleton et al., 2002). By incorporating phylogenetic information, we ensured that our conclusions reflect the true evolutionary relationships, reducing the risk of spurious correlations due to shared ancestry.

**Supplementary Figures**


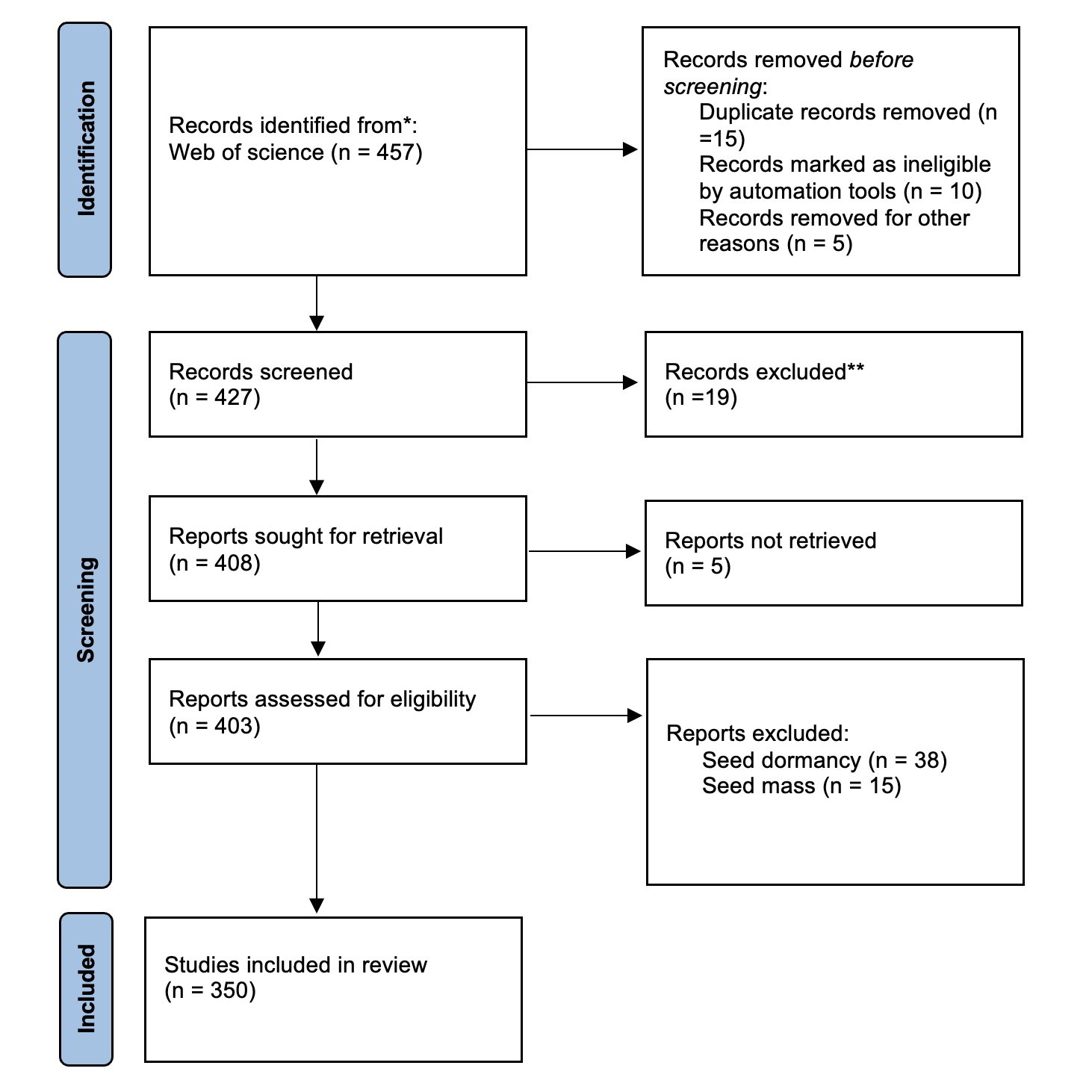


**Figure S1.** The PRISMA flow chart indicating an overview of literature search and categorization of reports.

**Fig S2.** The graph presented depicts the number of species included in the analysis of traits across various plant categories and dormancy types. The figures provided in the legend correspond to the total number of species represented in the graph.

**Figure S3.** The phylogenetic distribution of seed mass, embryo size ratio, and RLG. Colored areas represent trait values across the phylogeny, with each trait depicted using a different color gradient.

**Figure S4.** The frequency distribution of seed mass. Panels coded by (a) the frequency percentage of species grouped into different life forms, including trees, shrubs, and herbs across seed mass gradient (on a log scale) (b) the frequency percentage of species grouped into different dormancy types across seed mass gradient. (c) indicates the frequency distribution of seed mass for three different plant growth forms: trees, shrubs, and herbs, (d) shows the frequency distribution of seed mass for five different dormancy classes. The height of each curve represents the number of values collected and incorporated into the database, not the seed mass of each category. Instead, the magnitude of seed mass (mg) of each category is shown on the X-axis. Values in the parenthesis indicate the average value of seed mass in mg. PD: physiological dormancy. PY: physical dormancy. MD: morphological dormancy. MPD: morphophysiological dormancy. ND: non-dormancy.

**Fig S5.** Correspondence between seed mass and relative light germination in relation to life forms. Regression of relative light germination (RLG) on seed mass. Fitted lines were estimated via phylogenetic regression approach as suggested by Revell., 2010.

**Table S1. List of species used in this study**

| Species | Dormancy | Growth Form |
| --- | --- | --- |
| Acacia catechu | PY | Tree |
| Acacia dealbata | PY | Tree |
| Acacia nilotica | PY | Tree |
| Acacia melanoxylon | PY | Tree |
| Acacia decurrens | PY | Tree |
| Acacia auriculiformis | PY | Tree |
| Acacia sundra | PY | Tree |
| Acacia mosllisima | PY | Tree |
| Ailanthus excelsa | ND | Tree |
| Ailanthus malabaric | ND | Tree |
| Albizia stipulata | PY | Tree |
| Albizia odoratissim | PY | Tree |
| Albizia lrbbec | PY | Tree |
| Albizia amara | PY | Tree |
| Albizia molluccana | PY | Tree |
| Ahtonia scholaris | ND | Tree |
| Annona squamosa | MPD | Tree |
| Anthocephalus cadamba | ND | Tree |
| acrocarpus fraxinifolius | PY | Tree |
| artocarpus heterophyllus | ND | Tree |
| Artocarpus hirsutus | PD | Tree |
| Azadirachta indica | ND | Tree |
| Bombax ceiba | ND | Tree |
| borassus flabellifer | MPD | Tree |
| broussonetia papyrifera | ND | Tree |
| Buchanania lanzan | ND | Tree |
| calophyllum elatum | MPD | Tree |
| Canarium Strictum | PD | Tree |
| caryota urens | PD | Tree |
| cassia fistula | PY | Tree |
| cassia siamea | PY | Tree |
| casuarina equisetifolia | ND | Tree |
| Chloroxylon swietenia | ND | Tree |
| chukrasia tabularis | ND | Tree |
| cupressus torulosa | PY | Tree |
| dalbergia latifolia | PY | Tree |
| dalbergia sissoo | PY | Tree |
| dendrocalamus brandisii | ND | Tree |
| diospyros melanoxylon | ND | Tree |
| dipterocarpus indicus | PY | Tree |
| Duabuanga sonneratioides | ND | Tree |
| dysoxylum malabaricum | ND | Tree |
| Epidendrum paniculatum | ND | Orchid |
| elaeocarpus tuberculatus | PD | Tree |
| emblica officinalis | PD | Tree |
| erythrina indica | PY | Tree |
| eucalyptus citriodora | ND | Tree |
| evodia roxburghiana | ND | Tree |
| ficus infictotia | ND | Tree |
| Gmelina arborea | ND | Tree |
| grevillea robusta | ND | Tree |
| Grewia tiliaefolia | PY | Tree |
| hardwickia binata | ND | Tree |
| holoptelea integrifolia | ND | Tree |
| hopea wightiana | PD | Tree |
| hopea parviflora | PD | Tree |
| hymenodictyon excelsum | ND | Tree |
| kydia calycina | PY | Tree |
| lagerstroemia lanceolata | PD | Tree |
| leucaena leucocephala | PY | Tree |
| machilus macrantha | ND | Tree |
| madhuca indica | ND | Tree |
| Mangiifera indica | ND | Tree |
| macaranga peltata | MPD | Tree |
| melia dubia | MPD | Tree |
| Meospsis eminili | ND | Tree |
| michaelia champaca | MPD | Tree |
| michelia | MPD | Tree |
| mimusops elengi | PY | Tree |
| Melilotus alba | PY | Herb |
| myristica fragrans | PD | Tree |
| eugenia | ND | Tree |
| Palaguin ellipticum | ND | Tree |
| poeciloneuron indicum | PD | Tree |
| pongamia pinnata | ND | Tree |
| prosopis juliflora | PY | Tree |
| pterocarpus marsupium | PY | Tree |
| Pterocarpus santalinus | PD | Tree |
| Dipterocarpus alatus | ND | Tree |
| Samania saman | PY | Tree |
| Samanea saman | PY | Tree |
| shorea talura | ND | Tree |
| spondias tree | ND | Tree |
| sterculia campanulata | PD | Tree |
| sterculia alata | PY | Tree |
| Swietenia mahogany | ND | Tree |
| Symplocos spicata | MPD | Tree |
| Syzigium operculata | ND | Tree |
| Syzigium cumini | ND | Tree |
| terminalia tomentosa | MPD | Tree |
| terminalia paniculata | PY | Tree |
| terminalia belbicata | PY | Tree |
| terminalia arjuna | PD | Tree |
| Tetrameks nudajora | ND | Tree |
| vateria indica | PD | Tree |
| vateria indica | ND | Tree |
| Wrifhtia tinctoria | PY | Tree |
| krascheninnikovia lanata | PD | Herb |
| Potamogeton pectinatus | PD | Herb |
| Tropical tree | ND | Tree |
| Helianthus annuus | PD | Herb |
| Cicer arietinum | ND | Herb |
| Sugar Beet | PD | Herb |
| Uapaca kirkiana | PD | Tree |
| Mesua ferrea | ND | Tree |
| Clerodendrum bungei | PD | Tree |
| Triticum aestivum | PD | herb |
| cryptocarya alba | ND | Tree |
| Pinus taeda | PY | Tree |
| Albizia lebbek | PY | Tree |
| Tectona grandis | PD | Tree |
| Convolvulus arvensis | PD | herb |
| lychnis flos-cuculi | PD | Herb |
| Clematis microphylla | MD | Tree |
| Hedysarum laeve | PY | shrub |
| AVENA SATIVA | PD | Herb |
| Bellucia grossularioides | PD | Tree |
| Isertia hypoleuca | PD | shrub |
| Cecropia sciadophyla | PD | Tree |
| Jacaranda copaia | PD | Tree |
| Croton lanjowensis | PD | Tree |
| Byrsonima Byrsonima | PD | Shrub |
| G/ycine max | PD | herb |
| ctaea rubra (Ait.) Willd | PD | shrub |
| Rosa gymnocarpa Nutt. | PD | shrub |
| Sorbus scopulina Greene | PD | Tree |
| Symphoricarpos albus (L.) Blake | PY | shrub |
| Disporum trachycarpum | PY | herb |
| Smilacina racemosa | PY | Herb |
| Smilacina stellata | MPD | herb |
| Streptopus amplexifolius | MPD | Herb |
| sorbus domestica | PD | Tree |
| Magnolia kobus | MPD | Tree |
| Magnolia obovata | MPD | Tree |
| Elaeagnus umbellata | PD | shrub |
| Phellodendron amurense | PD | Tree |
| Alnus japonica | PD | Tree |
| Alnus hirsuta | PD | Tree |
| Betula ermanii | PD | Tree |
| Cercidiphyllum japonicum | PD | Tree |
| Betula platyphylla | PD | Tree |
| Hydrangea paniculata | PD | shrub |
| Lotus corniculstus | PY | Herb |
| Ligularia virgaurea | PD | shrub |
| Phyllanthus emblica | PD | Tree |
| Albizia procera | PD | Tree |
| Terminalia chebula | PD | Tree |
| Lespedeza davurica | PD | shrub |
| Pinus thunbergii | PD | tree |
| Elaeis guineensis | MD | tree |
| Pinus pinaster | PD | tree |
| Potamogeton pectinatus | ND | herb |
| Quercus griffit | ND | tree |
| Quercus dealbata | ND | tree |
| Peucedanum oreoselinum | MPD | herb |
| Leymus arenarius | PD | herb |
| Artemisia sieversiana Willd | ND | herb |
| Cremanthodium pleurocaule | PD | herb |
| Ligularia przewalskii | PD | herb |
| Senecio diversipinnus Ling | PD | herb |
| Saussurea hieracioides Hook | PD | herb |
| Saussurea iodostegia Hance | PD | herb |
| Saussurea japonica | ND | herb |
| Saussurea morifolia Chen | PD | herb |
| Saussurea mongolica(Franch.) | PD | herb |
| Saussurea parviflora (Poir.) DC | PD | herb |
| Saussurea variiloba Ling. | PD | herb |
| Tripolium vulgare Ness. | PD | herb |
| Ranunculus brotherusii var. tanguticus | MPD | herb |
| Trollius farreri Stapf | MPD | herb |
| Dracocephalum ruyschiana | PD | herb |
| Laniophlomis rotate (Benth.) | PD | herb |
| Salvia przewalskii Maxim | PD | herb |
| Hedysarum tanguticum Fedtsch | PY | herb |
| Medicago minima Bart | PY | herb |
| Vicia angustifolia L. | PY | herb |
| Cereus hankeanus K. Schumann | ND | Shrub |
| Cleistocactus baumannii (Lemaire) Lemaire | ND | Shrub |
| Echinopsis aurea Britton & Rose | PD | Shrub |
| Echinopsis leucantha (Salm-Dyck) Walpers | PD | Shrub |
| Echinopsis candicans (Salm-Dyck) Hunt | PD | Shrub |
| Echinopsis spiniflora (K. Schumann) Berger | PD | Shrub |
| Gymnocalycium bruchii (Spegazzini) Hosseus | ND | Shrub |
| Gymnocalycium capillense (Schick) Hosseus | PD | Shrub |
| Gymnocalycium castellanosii Backeb | ND | Shrub |
| Gymnocalycium monvillei (Lemaire) Britton & Rose | ND | Shrub |
| Gymnocalycium mostii (Gu¨ rke) Britton & Rose | ND | Shrub |
| Gymnocalycium quehlianum (F. Haage ex Quehl) Vaupel ex Hosseus | ND | Shrub |
| Gymnocalycium schickendantzii (F.A.C. Weber) Britton & Rose | ND | Shrub |
| Gymnocalycium stellatum Speg | ND | Shrub |
| Harrisia pomanensis (F.A.C. Weber) Britton & Rose | PD | Shrub |
| Parodia mammulosa (Lemaire) Taylor | PD | Shrub |
| Stetsonia coryne (Salm-Dyck) Britton & Rose | PD | Shrub |
| Agrostis alba | ND | herb |
| Agrostis hugouiana | PD | herb |
| Agrostis stolonifera | PD | herb |
| Poa poophagorum | PD | herb |
| Poa botryoides | PD | herb |
| Poa schoenites | PD | herb |
| Helictotrichon schellianum | PD | herb |
| Deyeuxia arundinacea | ND | herb |
| Ptilagrostis dichotoma var. roshevitsiana | PY | herb |
| Koeleria cristata | ND | herb |
| Deschampsia caespitosa | PD | herb |
| Poa declinata | PD | herb |
| Poa pratensis | PD | herb |
| Agrostis trinii | PD | herb |
| Helictotrichon leianthum | PD | herb |
| Mellica scabrosa | PD | herb |
| Ptilagrostis junatovii | PD | herb |
| Festuca ovina | PD | herb |
| Orinus kokonorica | PD | herb |
| Deyeuxia scabrescens | PD | herb |
| Festuca rubra | PD | herb |
| Poa attenuate var. vivipara | PD | herb |
| Festuca sinensis | PD | herb |
| Achnatherum splendens | ND | herb |
| Ptilagrostis dichotoma | PY | herb |
| Achnatherum inebrians | ND | herb |
| Ptilagrostis mongholica | PD | herb |
| Stipa purpurea | ND | herb |
| Stipa aliena | ND | herb |
| Helictotrichon tibeticum | PD | herb |
| Oryzopsis munroi | PD | herb |
| Achnatherum sibiricum | ND | herb |
| Elymus dahuricus | ND | herb |
| Bromus magnus | PD | herb |
| Bromus tectorum | PD | herb |
| Roegneria stricta | PD | herb |
| Brachypodium sylvaticum var. gracile | PD | herb |
| Roegneria kokonorica | PD | herb |
| Brachypodium sylvaticum | PD | herb |
| Bromus sinensis | PD | herb |
| Stipa capillacea | ND | herb |
| Stipa przewalskyi | ND | herb |
| Aneurolepidium dasystachys | PD | herb |
| Elymus tangutorum | PD | herb |
| Roegneria nutans | PD | herb |
| Aristida triseta | PD | herb |
| Bacharis medullosa | PD | shrub |
| Bacharis dracunculifolia | ND | shrub |
| Eupatorium buniifolium | PD | shrub |
| Parthenium hysterophorus | PD | tree |
| Zinnia peruviana | PD | tree |
| Flourensia campestris | PD | tree |
| Flourensia oolepis | PD | tree |
| Abarema jupunba | ND | tree |
| Acacia farnesiana | PY | tree |
| Acacia_polyphylla | PY | tree |
| Acrocomia aculeata | MPD | tree |
| Agonandra brasiliensis | MPD | tree |
| Albizia inundata | PY | tree |
| Albizia_niopoides | PY | tree |
| Albizia_tubulosa | PY | tree |
| Alchornea triplinervia | PD | tree |
| Alexa grandiflora | ND | tree |
| Allophylus edulis | ND | tree |
| Alseis floribunda | PD | tree |
| Amaioua guianensis | PD | tree |
| Ambelania acida | PD | tree |
| Amburana cearensis | ND | tree |
| Anacardium giganteum | ND | tree |
| Anacardium occidentale | PD | tree |
| Anadenanthera colubrina | ND | tree |
| Anadenanthera peregrina | ND | tree |
| Andira inermis | PD | tree |
| Aniba firmula | PD | tree |
| Aniba_rosaeodora | PD | tree |
| Apeiba_tibourbou | PY | tree |
| Apuleia_leiocarpa | PY | tree |
| Aspidosperma_cylindrocarpon | PY | tree |
| Aspidosperma_discolor | PY | tree |
| Aspidosperma_macrocarpon | PY | tree |
| Aspidosperma_parvifolium | PY | tree |
| Aspidosperma_polyneuron | PY | tree |
| Aspidosperma_spruceanum | PY | tree |
| Astrocaryum_vulgare | ND | tree |
| Astronium_fraxinifolium | ND | tree |
| Astronium_graveolens | PY | tree |
| Bactris_gasipaes | PY | tree |
| Bagassa_guianensis | PY | tree |
| Balizia_pedicellaris | PY | tree |
| Banara_arguta | PY | tree |
| Bauhinia_longifolia | ND | tree |
| Bertholletia_excelsa | MPD | tree |
| Bixa_arborea | ND | tree |
| Bixa_orellana | PY | tree |
| Bocageopsis_multiflora | ND | tree |
| Bougainvillea_glabra | PD | Shrub |
| Bowdichia_virgilioides | ND | tree |
| Brosimum_gaudichaudii | ND | tree |
| Brosimum_guianense | MPD | tree |
| Brownea_grandiceps | ND | tree |
| Buchenavia_tomentosa | ND | tree |
| Byrsonima_coccolobifolia | MPD | tree |
| Byrsonima_sericea | PD | Shrub |
| Byrsonima_spicata | PD | tree |
| Byrsonima_stipulacea | PY | tree |
| Callisthene_fasciculata | PY | tree |
| Calophyllum_brasiliensis | ND | tree |
| Calycophyllum_spruceanum | ND | tree |
| Campomanesia_guazumifolia | ND | tree |
| Campomanesia_xanthocarpa | PY | tree |
| Carapa_guianensis | PY | tree |
| Cardiopetalum_calophyllum | PY | tree |
| Cariniana_estrellensis | ND | tree |
| Caryocar_brasiliense | ND | tree |
| Caryocar_microcarpum | PY | tree |
| Caryocar_villosum | ND | tree |
| Casearia_decandra | ND | tree |
| Casearia_sylvestris | ND | tree |
| Cassia_grandis | PD | tree |
| Cassia_leiandra | PD | tree |
| Cecropia_hololeuca | PY | tree |
| Cecropia_pachystachya | ND | tree |
| Cecropia_purpurascens | ND | tree |
| Cecropia_sciadophylla | ND | tree |
| Cedrela_fissilis | ND | tree |
| Cedrela_odorata | ND | tree |
| Ceiba_pentandra | PY | tree |
| Ceiba_samauma | ND | tree |
| Celtis_iguanea | ND | tree |
| Cenostigma_macrophyllum | ND | tree |
| Cenostigma_tocantinum | PD | tree |
| Chimarrhis_barbata | PD | tree |
| Chorisia_speciosa | ND | tree |
| Chrysobalanus_icaco | PY | tree |
| Chrysophyllum_gonocarpum | PD | tree |
| Clitoria_fairchildiana | PY | tree |
| Coccoloba_mollis | ND | tree |
| Cocos_nucifera | ND | tree |
| Colubrina_glandulosa | ND | tree |
| Combretum_leprosum | MPD | tree |
| Commiphora_leptophloeos | MPD | tree |
| Copaifera_langsdorffii | ND | tree |
| Cordia_alliodora | MPD | tree |
| Cordia_goeldiana | MPD | tree |
| Cordia_sellowiana | PY | tree |
| Couma_utilis | PY | tree |
| Couroupita_guianensis | PD | tree |
| Coutarea_hexandra | ND | tree |
| Crataeva_tapia | ND | tree |
| Croton_lanjouwensis | PD | tree |
| Cupania_vernalis | ND | tree |
| Curatella_americana | PY | tree |
| Cybistax_antisyphilitica | PY | tree |
| Dendropanax_cuneatus | PD | tree |
| Dialium_guianense | ND | tree |
| Dilodendron_bipinnatum | PY | tree |
| Dimorphandra_macrostachya | PY | tree |
| Dinizia_excelsa | ND | tree |
| Diospyros_inconstans | ND | tree |
| Diplokeleba_floribunda | PD | tree |
| Diplotropis_purpurea | PY | tree |
| Dipteryx_alata | ND | tree |
| Dipteryx_odorata | MPD | tree |
| Diptychandra_aurantiaca | ND | tree |
| Dodonaea_viscosa | ND | tree |
| Drimys_winteri | MPD | tree |
| Duckeodendron_cestroides | PY | tree |
| Emmotum_nitens | PY | tree |
| Endlicheria_paniculata | PD | tree |
| Endopleura_uchi | ND | tree |
| Enterolobium_contortisiliquum | PD | tree |
| Enterolobium_schomburgkii | ND | tree |
| Erisma_uncinatum | PY | tree |
| Erythrina_crista-galli | PD | tree |
| Erythrina_fusca | PD | tree |
| Erythrina_poeppigiana | PD | tree |
| Erythrina_verna | PD | tree |
| Eschweilera_coriacea | PD | tree |
| Eschweilera_ovata | PD | tree |
| Esenbeckia_grandiflora | PD | tree |
| Eugenia_brasiliensis | PD | tree |
| Eugenia_florida | PD | tree |
| Eugenia_uniflora | PD | tree |
| Euterpe_oleracea | PD | tree |
| Ficus_calyptroceras | PD | tree |
| Ficus_catappifolia | ND | tree |
| Ficus_dendrocida | ND | tree |
| Ficus_enormis | ND | tree |
| Ficus_insipida | ND | tree |
| Gallesia_integrifolia | ND | tree |
| Garcinia_gardneriana | PD | tree |
| Genipa_americana | PD | tree |
| Gomidesia_lindeniana | ND | tree |
| Goupia_glabra | ND | tree |
| Guapira_opposita | ND | tree |
| Guarea_guidonia | ND | tree |
| Guarea_kunthiana | ND | tree |
| Guatteria_citriodora | ND | tree |
| Guazuma_crinita | ND | tree |
| Guazuma_ulmifolia | ND | tree |
| Guettarda_viburnoides | ND | tree |
| Gustavia_augusta | PD | tree |
| Heliocarpus_americanus | PD | tree |
| Hevea_brasiliensis | PD | tree |
| Hibiscus_pernambucensis | PD | tree |
| Hieronima_alchorneoides | PD | tree |
| Himatanthus_obovatus | PD | tree |
| Hirtella_ciliata | PD | tree |
| Hirtella_glandulosa | PD | tree |
| Hirtella_hebeclada | PD | tree |
| Holocalyx_balansae | PD | tree |
| Humiria_balsamifera | PD | tree |
| Hura_crepitans | PD | tree |
| Ilex_affinis | PD | tree |
| Ilex_theezans | PD | tree |
| Inga_cylindrica | PD | tree |
| Inga_edulis | PD | tree |
| Inga_laurina | PD | tree |
| Inga_marginata | PD | tree |
| Inga_vera | PD | tree |
| Isertia_hypoleuca | PD | tree |
| Jacaranda_copaia | PD | tree |
| Jacaratia_spinosa | ND | tree |
| Lecythis_chartacea | ND | tree |
| Lecythis_latifolium | PD | tree |
| Lecythis_lurida | PD | tree |
| Lecythis_pisonis | PD | tree |
| Licania_parvifolia | PD | tree |
| Lithraea_molleoides | PD | tree |
| Lonchocarpus_araripensis | ND | tree |
| Lonchocarpus_guillemineanus | ND | tree |
| Lonchocarpus_sericeus | ND | tree |
| Lophanthera_lactescens | PY | tree |
| Luehea_candicans | PY | tree |
| Luehea_grandiflora | PY | tree |
| Luehea_paniculata | PY | tree |
| Mabea_fistulifera | PY | tree |
| Machaerium_aculeatum | PY | tree |
| Machaerium_acutifolium | PY | tree |
| Machaerium_nyctitans | PY | tree |
| Machaerium_villosum | PY | tree |
| Maclura_tinctoria | PY | tree |
| Macrolobium_bifolium | PY | tree |
| Magnolia_ovata | PY | tree |
| Magonia_pubescens | PY | tree |
| Manilkara_huberi | PY | tree |
| Maprounea_guianensis | PY | tree |
| Margaritaria_nobilis | PY | tree |
| Mauritia_flexuosa | PD | tree |
| Mezilaurus_itauba | PD | tree |
| Micrandra_elata | PD | tree |
| Mimosa_tenuiflora | PD | tree |
| Minquartia_guianensis | PD | tree |
| Myrcia_rostrata | PD | tree |
| Myrcia_tomentosa | PD | tree |
| Myrocarpus_frondosus | PD | tree |
| Myroxylon_peruiferum | PD | tree |
| Nectandra_cissiflora | PD | tree |
| Nectandra_megapotamica | PD | tree |
| Nectandra_membranacea | PD | tree |
| Nectandra_nitidula | PD | tree |
| Nectandra_rigida | PD | tree |
| Ochroma_pyramidale | PD | tree |
| Ocotea_puberula | PD | tree |
| Ocotea_pulchella | PD | tree |
| Orbignya_speciosa | PD | tree |
| Ormosia_arborea | PD | tree |
| Pachira_aquatica | PD | tree |
| Parkia_multijuga | PD | tree |
| Parkia_nitida | PD | tree |
| Parkia_pendula | PD | tree |
| Parkia_platycephala | PD | tree |
| Parkinsonia_aculeata | PD | tree |
| Peltogyne_angustiflora | MD | tree |
| Peltophorum_dubium | MD | tree |
| Pentaclethra_macroloba | MD | tree |
| Pera_glabrata | PY | tree |
| Physocalymma_scaberrimum | PY | tree |
| Phytolacca_dioica | PY | tree |
| Piptadenia_gonoacantha | PD | tree |
| Platonia_insignis | PD | tree |
| Platypodium_elegans | PD | tree |
| Polyandrococos_caudescens | PD | tree |
| Poraqueiba_sericea | PD | tree |
| Pourouma_cecropiifolia | PD | tree |
| Pourouma_guianensis | PD | tree |
| Pouteria_caimito | PD | tree |
| Pouteria_macrophylla | PD | tree |
| Pouteria_ramiflora | PD | tree |
| Pouteria_torta | PD | tree |
| Pouteria_venosa | PD | tree |
| Protium_heptaphyllum | PD | tree |
| Protium_spruceanum | PD | tree |
| Pseudobombax_grandiflorum | PD | tree |
| Pseudobombax_marginatum | PD | tree |
| Psidium_guajava | PD | tree |
| Psidium_sartorianum | PY | tree |
| Pterodon_emarginatus | PY | tree |
| Qualea_grandiflora | PY | tree |
| Qualea_paraensis | MPD | tree |
| Qualea_parviflora | MPD | tree |
| Rapanea_ferruginea | PD | tree |
| Rapanea_umbellata | PD | tree |
| Rollinia_sylvatica | PD | tree |
| Rudgea_viburnoides | MPD | tree |
| Sacoglottis_guianensis | MPD | tree |
| Salacia_elliptica | PD | tree |
| Salix_humboldtiana | PD | tree |
| Sapindus_saponaria | PD | tree |
| Sapium_glandulatum | PD | tree |
| Scheelea_phalerata | PD | tree |
| Schefflera_morototoni | PD | tree |
| Schinus_terebinthifolia | PD | tree |
| Schistostemon_retusum | PD | tree |
| Schizolobium_parahyba | PY | tree |
| Sciadodendron_excelsum | PY | tree |
| Sclerolobium_paniculatum_var.rubiginosum | PY | tree |
| Sclerolobium_rugosum | PY | tree |
| Senna_multijuga | PY | tree |
| Senna_spectabilis | PD | tree |
| Simarouba_amara | PD | tree |
| Sloanea_monosperma | PD | tree |
| Solanum_lycocarpum | PD | tree |
| Sparattosperma_leucanthum | PD | tree |
| Spondias_mombin | PD | tree |
| Spondias_venulosa | PY | tree |
| Sterculia_apetala | PY | tree |
| Sterculia_chicha | PD | tree |
| Sterculia_striata | PD | tree |
| Stryphnodendron_pulcherrimum | PD | tree |
| Sweetia_fruticosa | PD | tree |
| Swietenia_macrophylla | PD | tree |
| Symphonia_globulifera | PD | tree |
| Tabebuia_aurea | PD | tree |
| Tabebuia_impetiginosa | PD | tree |
| Tabebuia_insignis | PD | tree |
| Tabebuia_ochracea | PD | tree |
| Tabebuia_roseo-alba | PD | tree |
| Tabebuia_serratifolia | PD | tree |
| Tabebuia_vellosoi | PD | tree |
| Tachigali_multijuga | PD | tree |
| Talisia_esculenta | PD | tree |
| Tapirira_guianensis | PD | tree |
| Tapirira_obtusa | PD | tree |
| Terminalia_argentea | PD | tree |
| Terminalia_fagifolia | PD | tree |
| Terminalia_lucida | PD | tree |
| Theobroma_cacao | PD | tree |
| Theobroma_grandiflorum | PD | tree |
| Theobroma_speciosum | PD | tree |
| Tibouchina_granulosa | PD | tree |
| Toulicia_guianensis | PD | tree |
| Trattinickia_rhoifolia | PD | tree |
| Trichilia_hirta | PD | tree |
| Trichilia_pallida | PD | tree |
| Triplaris_americana | PD | tree |
| Triplaris_surinamensis | PD | tree |
| Vantanea_parviflora | PD | tree |
| Virola_sebifera | MD | tree |
| Virola_surinamensis | MD | tree |
| Vitex_cymosa | MD | tree |
| Vochysia_haenkeana | PD | tree |
| Vochysia_tucanorum | PD | tree |
| Vouacapoua_americana | PD | tree |
| Warszewiczia_coccinea | PD | tree |
| Xylopia_brasiliensis | PD | tree |
| Xylopia_emarginata | PD | tree |
| Xylopia_frutescens | PD | tree |
| Xylopia_sericea | PD | tree |
| Zanthoxylum_rhoifolium | PD | tree |
| Zanthoxylum_riedelianum | PD | tree |
| Zeyheria_tuberculosa | PD | tree |
| Acacia_auriculiformis | PD | tree |
| Acacia_catecha | PD | tree |
| Acacia_dealhata | PD | tree |
| Acacia_decurrens | PD | tree |
| Acacia_melanoxylon | PD | tree |
| Acacia_mosllisima | PD | tree |
| Acacia_nilotica | ND | tree |
| Acacia_sundra | ND | tree |
| Ailanthus_excelsa | ND | tree |
| Ailanthus_malaharica | ND | tree |
| Albizia_amara | ND | tree |
| Albizia_lebbec | ND | tree |
| Albizia_molluccana | ND | tree |
| Albizia_odoratissima | MPD | tree |
| Albizia_saman | MPD | tree |
| Albizia_stipulosa | MPD | tree |
| Alstonia_scholaris | MPD | tree |
| Annona_squamosa | MPD | tree |
| Artocarpus_heterophylla | MPD | tree |
| Artocarpus_hirsusa | MPD | tree |
| Azadirachta_indica | MPD | tree |
| Bombax_ceiba | PD | tree |
| Borassus_flabellifera | PD | tree |
| Breonia_kadamba | PD | tree |
| Broussonetia_papyrifera | PD | tree |
| Buchanania_lanzan | PD | tree |
| Calophyllum_elatum | PD | tree |
| Canarium_strictum | PD | tree |
| Caryota_urens | PD | tree |
| Cassia_fistula | PD | tree |
| Cassia_siamea | PD | tree |
| Casuarina_equisetifolia | PD | tree |
| Chloroxylon_swietenia | PD | tree |
| Chukrasia_tabularis | PD | tree |
| Cupressus_torulosa | PD | tree |
| Dalbergia_latifolia | PD | tree |
| Dalbergia_sissoo | PD | tree |
| Dendrocalamus_brandisi | PD | tree |
| Desmodium_dalbergioides | PD | tree |
| Diospyros_melanoxylon | ND | tree |
| Dipterocarpus_indicus | PD | tree |
| Duabanga_sonneratioides | PD | tree |
| Dysoxylum_malabaricum | PD | tree |
| Elaeocarpus_tuberculatus | PD | tree |
| Elaeodendron_paniculatum | PD | tree |
| Erythrina_indica | ND | tree |
| Eucalyptus_citriodora | PD | tree |
| Euodia_roxburgiana | PD | tree |
| Ficus_infectoria | PD | tree |
| Gmelina_arborea | PD | tree |
| Grevillea_robusta | PD | tree |
| Grewia_tillaefolia | MPD | tree |
| Hardwickia_binata | MPD | tree |
| Hardwickia_pinnata | PD | tree |
| Holoptelea_integrifolia | PD | tree |
| Hopea_parviflora | PD | tree |
| Hopea_wightiana | PY | tree |
| Hymenodictyon_excelsum | PY | tree |
| Kydia_calycina | PY | tree |
| Lagerstroemia_lanceolasa | ND | tree |
| Leucaena_leucocephala | ND | tree |
| Macaranga_peltata | PD | tree |
| Madhuca_indica | PD | tree |
| Mangifera_indica | PD | tree |
| Melia_dubia | PD | tree |
| Michelia_champaca | ND | tree |
| Michelia_doltsopa | PD | tree |
| Mimusops_elengi | ND | tree |
| Morus_alba | ND | tree |
| Myristica_fragens | ND | tree |
| Palaquium_ellipticum | ND | tree |
| Persea_macarantha | ND | tree |
| Phyllanthus_officinalis | ND | tree |
| Poeciloneuron_indicum | PD | tree |
| Pongamia_pinnata | PD | tree |
| Prosopis_julifora | PD | tree |
| Pterocarpus_dalbergioides | ND | tree |
| Pterocarpus_marsupium | PD | tree |
| Pterocarpus_santalinus | PD | tree |
| Santalum_album | PD | tree |
| Shorea_talura | PD | tree |
| Spondias_trifoliatus | PD | tree |
| Sterculia_alata | PD | tree |
| Sterculia_companulata | ND | tree |
| Swietenia_mahogany | PY | tree |
| Symplocos_spicata | ND | tree |
| Syzygium_cumini | PD | tree |
| Syzygium_operculatum | PD | tree |
| Terminalia_arjuna | PD | tree |
| Terminalia_bellerica | PD | tree |
| Terminalia_paniculata | PD | tree |
| Terminalia_tomentosa | PD | tree |
| Tetrameles_nudiflora | PD | tree |
| Vateria_indica | PD | tree |
| Vateria_sp. | PD | tree |
| Wrightia_tinctoria | PD | tree |
| Acacia_decurrens | ND | tree |
| Acacia_melanoxylon | PY | tree |
| Acacia_mosllisima | ND | tree |
| Acacia_nilotica | PD | tree |
| Albizia_amara | PD | tree |
| Albizia_lebbec | PD | tree |
| Albizia_saman | ND | tree |
| Albizia_stipulosa | PD | tree |
| Alstonia_scholaris | PD | tree |
| Annona_squamosa | PD | tree |
| Artocarpus_heterophylla | PD | tree |
| Artocarpus_hirsusa | PD | tree |
| Azadirachta_indica | PD | tree |
| Borassus_flabellifera | ND | tree |
| Breonia_kadamba | ND | tree |
| Cassia_fistula | ND | tree |
| Dalbergia_latifolia | ND | tree |
| Dalbergia_sissoo | PY | tree |
| Dendrocalamus_brandisi | PY | tree |
| Desmodium_dalbergioides | MPD | tree |
| Diospyros_melanoxylon | MPD | tree |
| Dipterocarpus_indicus | PY | tree |
| Duabanga_sonneratioides | PY | tree |
| Dysoxylum_malabaricum | PY | tree |
| Elaeodendron_paniculatum | ND | tree |
| Gmelina_arborea | ND | tree |
| Grevillea_robusta | ND | tree |
| Grewia_tillaefolia | PD | tree |
| Hardwickia_pinnata | ND | tree |
| Holoptelea_integrifolia | ND | tree |
| Hopea_wightiana | PD | tree |
| Hymenodictyon_excelsum | PD | tree |
| Lagerstroemia_lanceolasa | PY | tree |
| Macaranga_peltata | PY | tree |
| Madhuca_indica | PY | tree |
| Mangifera_indica | PY | tree |
| Melia_dubia | PY | tree |
| Michelia_champaca | PY | tree |
| Morus_alba | PY | tree |
| Persea_macarantha | PY | tree |
| Phyllanthus_officinalis | PY | tree |
| Poeciloneuron_indicum | PY | tree |
| Pongamia_pinnata | PY | tree |
| Prosopis_julifora | PY | tree |
| Pterocarpus_marsupium | MPD | tree |
| Pterocarpus_santalinus | ND | tree |
| Santalum_album | PY | tree |
| Shorea_talura | ND | tree |
| Sterculia_alata | ND | tree |
| Swietenia_mahogany | MPD | tree |
| Syzygium_cumini | ND | tree |
| Syzygium_operculatum | MPD | tree |
| Terminalia_tomentosa | PY | tree |
| Tetrameles_nudiflora | PY | tree |
| Vateria_indica | ND | tree |
| Vateria_sp. | ND | tree |
| Wrightia_tinctoria | ND | tree |
| Acioa_barteri | PY | tree |
| Acioa_scabrifolia | PY | tree |
| Afraegle_paniculata | PY | tree |
| Afzelia_africana | ND | tree |
| Afzelia_bella | ND | tree |
| Albizia_adianthifolia | PY | tree |
| Albizia_altissima | ND | tree |
| Albizia_coriara | ND | tree |
| Albizia_dinklagei | ND | tree |
| Albizia_ferruginea | PD | tree |
| Albizia_glaberrima | PD | tree |
| Albizia_zygia | PY | tree |
| Allophylus_africanus | ND | tree |
| Alstonia_congensis | ND | tree |
| Amphimas_pterocarpoides | ND | tree |
| Annickia_polycarpa | ND | tree |
| Anopyxis_klaineana | ND | tree |
| Anthonotha_crassifolia | PY | tree |
| Anthonotha_fragans | ND | tree |
| Anthonotha_macrophylla | ND | tree |
| Anthonotha_vignei | ND | tree |
| Anthostema_aubryanum | PD | tree |
| Antiaris_africana | PD | tree |
| Antiaris_welwitschii | ND | tree |
| Antrocaryon_micraster | PY | tree |
| Aubregrinia_taiensis | PD | tree |
| Aubrevillea_platycarpa | PY | tree |
| Aucoumea_klainei | ND | tree |
| Balanites_wilsoniana | ND | tree |
| Baphia_bancoensis | ND | tree |
| Baphia_nitida | MPD | tree |
| Beilschmiedia_mannii | MPD | tree |
| Beilschmiedia_sp | ND | tree |
| Berlinia_confusa | MPD | tree |
| Berlinia_occidentalis | MPD | tree |
| Bersama_paullinoides | PY | tree |
| Blighia_sapida | PY | tree |
| Blighia_unijugata | PD | tree |
| Blighia_welwitschii | ND | tree |
| Bombax_brevicuspe | ND | tree |
| Bombax_buonopozense | PD | tree |
| Breviea_sericea | ND | tree |
| Bridelia_atroviridis | PY | tree |
| Bridelia_aubrevillei | PY | tree |
| Buchholzia_coriacea | PD | tree |
| Bussea_occidentalis | ND | tree |
| Caloncoba_brevipes | PY | tree |
| Caloncoba_echinata | PY | tree |
| Calpocalyx_aubrevillei | ND | tree |
| Calpocalyx_brevibracteatus | ND | tree |
| Canarium_sweinfurthii | PD | tree |
| Canthium_subcordatum | PY | tree |
| Canthium_tekbe | ND | tree |
| Carapa_procera | MPD | tree |
| Carpolobia_lutea | ND | tree |
| Cassia_fikifiki | ND | tree |
| Cassia_sieberiana | MPD | tree |
| Cassipourea_sp | PY | tree |
| Celtis_adolfifriderici | PD | tree |
| Celtis_mildbraedii | ND | tree |
| Chidlowia_sanguinea | PD | tree |
| Chionanthus_lingelsheimiana | ND | tree |
| Chrysophyllum_africanum | PY | tree |
| Chrysophyllum_beguei | PD | tree |
| Chrysophyllum_giganteum | PD | tree |
| Chrysophyllum_perpulchrum | PD | tree |
| Chrysophyllum_pruniforme | PD | tree |
| Chrysophyllum_subnudum | PD | tree |
| Chrysophyllum_taiensis | PD | tree |
| Cleistopholis_patens | PD | tree |
| Coelocaryon_oxycarpum | PD | tree |
| Cola_gigantea | PD | tree |
| Cola_lateritia | PD | tree |
| Cola_nitida | PD | tree |
| Copaifera_salikounda | PD | tree |
| Cordia_platythyrsa | ND | tree |
| Cordia_senegalensis | ND | tree |
| Coula_edulis | ND | tree |
| Croton_aubrevillei | ND | tree |
| Croton_zambesicus | ND | tree |
| Crudia_gabonensis | PD | tree |
| Crudia_klainei | PD | tree |
| Crudia_senegalensis | ND | tree |
| Cussonia_bancoensis | ND | tree |
| Cylicodiscus_gabonensis | ND | tree |
| Cynometra_ananta | ND | tree |
| Cynometra_megalophylla | ND | tree |
| Dacryodes_klaineana | ND | tree |
| Daniellia_thurifera | ND | tree |
| Deinbollia_pinnata | ND | tree |
| Desplatsia_chrysochlamys | ND | tree |
| Desplatsia_subericarpa | PD | tree |
| Detarium_senegalense | PD | tree |
| Dialium_aubrevillei | PD | tree |
| Dialium_dinklagei | PD | tree |
| Dialium_guineense | PD | tree |
| Dichapetalum_guineense | PD | tree |
| Diospyros_gabonensis | PD | tree |
| Diospyros_ivorensis | PD | tree |
| Diospyros_kamerunensis | PD | tree |
| Diospyros_mannii | PD | tree |
| Diospyros_sanzaminica | PD | tree |
| Diospyros_soubreana | PD | tree |
| Distemonanthus_benthamianus | PD | tree |
| Dracaena_mannii | PD | tree |
| Drypetes_aylmeri | PD | tree |
| Drypetes_klainnei | PD | tree |
| Duboscia_viridiflora | PD | tree |
| Ekebergia_senegalensis | PD | tree |
| Elaeophorbia_grandifolia | PD | tree |
| Entandrophragma_angolense | PD | tree |
| Entandrophragma_candollei | PD | tree |
| Entandrophragma_cylindricum | ND | tree |
| Entandrophragma_utile | ND | tree |
| Eriocoelum_pungens | PD | tree |
| Erythrina_mildbraedii | PD | tree |
| Erythrina_vogelii | PD | tree |
| Erythrophleum_ivorense | PD | tree |
| Erythroxylum_mannii | PD | tree |
| Funtumia_elastica | ND | tree |
| Funtumia_latifolia | ND | tree |
| Garcinia_gnetoides | ND | tree |
| Garcinia_kola | PY | tree |
| Garcinia_polyantha | PY | tree |
| Gilbertiodendron_limba | PY | tree |
| Gilbertiodendron_splendidum | PY | tree |
| Gilbertiodendron_taiense | PY | tree |
| Gilletiodendron_kisantuense | PY | tree |
| Gluema_ivorensis | PY | tree |
| Guarea_cedrata | PY | tree |
| Guarea_thompsonii | PY | tree |
| Guibourtia_ehie | PY | tree |
| Gymnostemon_zaizou | PY | tree |
| Hannoa_klaineana | PY | tree |
| Hemandradenia_chevalieri | PY | tree |
| Heritiera_utilis | PY | tree |
| Hexalobus_crispiflorus | PY | tree |
| Hildegardia_barteri | PY | tree |
| Holarrhena_africana | PD | tree |
| Holoptelea_grandis | PD | tree |
| Hoplestigma_klaineanum | PD | tree |
| Hunteria_eburnea | PD | tree |
| Hymenostegia_afzelii | PD | tree |
| Hymenostegia_aubrevillei | PD | tree |
| Irvingia_gabonensis | PD | tree |
| Irvingia_ivorensis | PD | tree |
| Keayodendron_bridelioides | PD | tree |
| Khaya_anthotheca | PD | tree |
| Khaya_grandifolia | PD | tree |
| Khaya_ivorensis | PD | tree |
| Kigelia_tristis | PD | tree |
| Lannea_welwitschii | PD | tree |
| Lecaniodiscus_cupanioides | PD | tree |
| Leptonychia_urophylla | PD | tree |
| Lindackeria_dentata | PD | tree |
| Lonchocarpus_sericeus | PD | tree |
| Lophira_alata | PD | tree |
| Lovoa_klaineana | PD | tree |
| Macaranga_barteri | PD | tree |
| Macaranga_spinosa | PD | tree |
| Maclura_excelsa | PD | tree |
| Maesopsis_eminii | PD | tree |
| Majidea_fosteri | PD | tree |
| Mammea_africana | MD | tree |
| Manilkara_lacera | MD | tree |
| Manilkara_sylvestris | MD | tree |
| Mansonia_altissima | PY | tree |
| Markhamia_tomentosa | PY | tree |
| Memecylon_cinnamomoides | PY | tree |
| Millettia_zechiana | PD | tree |
| Monodora_myristica | PD | tree |
| Monodora_tenuifolia | PD | tree |
| Morinda_lucida | PD | tree |
| Morus_mesozygia | PD | tree |
| Myrianthus_arboreus | PD | tree |
| Nesogordonia_papaverifera | PD | tree |
| Newbouldia_laevis | PD | tree |
| Newtonia_aubrevillei | PD | tree |
| Newtonia_duparquetiana | PD | tree |
| Ochna_multiflora | PD | tree |
| Octoknema_borealis | PD | tree |
| Okoubaka_aubrevillei | PD | tree |
| Oldfieldia_africana | PD | tree |
| Omphalocarpum_ahia | PD | tree |
| Omphalocarpum_anocentrum | PD | tree |
| Omphalocarpum_pachysteloides | PD | tree |
| Ongokea_gore | PY | tree |
| Ophiobotrys_zenkeri | PY | tree |
| Oricia_suaveolens | PY | tree |
| Ouratea_sp | MPD | tree |
| Pachypodanthium_staudtii | MPD | tree |
| Panda_oleosa | PD | tree |
| Parinari_chrysophylla | PD | tree |
| Parinari_congensis | PD | tree |
| Parinari_excelsa | MPD | tree |
| Parinari_glabra | MPD | tree |
| Parinari_holstii | PD | tree |
| Parkia_bicolor | PD | tree |
| Pentaclethra_macrophylla | PD | tree |
| Pentadesma_butyracea | PD | tree |
| Pericopsis_elata | PD | tree |
| Petersianthus_africanum | PD | tree |
| Phyllanthus_discoideus | PD | tree |
| Picralima_nitida | PD | tree |
| Piptadeniastrum_africanum | PY | tree |
| Placodiscus_bancoensis | PY | tree |
| Placodiscus_boya | PY | tree |
| Plagiosiphon_emarginatus | PY | tree |
| Pleiocarpa_mutica | PY | tree |
| Pouteria_micrantha | PD | tree |
| Protomegabaria_stapfiana | PD | tree |
| Pseudospondias_microcarpa | PD | tree |
| Psychotria_venosa | PD | tree |
| Pteleopsis_hylodendron | PD | tree |
| Pterocarpus_santalinoides | PD | tree |
| Pterygota_bequartii | PY | tree |
| Pterygota_macrocarpa | PY | tree |
| Pycnanthus_angolensis | PD | tree |
| Rauvolfia_vomitora | PD | tree |
| Ricinodendron_heudelotii | PD | tree |
| Rinorea_longicuspis | PD | tree |
| Sacoglottis_gabonensis | PD | tree |
| Sapium_aubrevillei | PD | tree |
| Scaphopetalum_amoenum | PD | tree |
| Schrebera_arborea | PD | tree |
| Scottellia_chevalieri | PD | tree |
| Scottellia_coriacea | PD | tree |
| Scytopetalum_tieghemii | PD | tree |
| Spathodea_campanulata | PD | tree |
| Spondianthus_preussii | PD | tree |
| Stemonocoleus_micranthus | PD | tree |
| Sterculia_oblonga | PD | tree |
| Sterculia_rhinopetala | PD | tree |
| Sterculia_tragacantha | PD | tree |
| Stereospermum_acuminatissimum | PD | tree |
| Strephonema_pseudocola | PD | tree |
| Strombosia_glaucescens | PD | tree |
| Swartzia_fistuloides | PD | tree |
| Synsepalum_brevipes | PD | tree |
| Syzygium_littorale | PD | tree |
| Syzygium_rowlandii | PD | tree |
| Tabernaemontana_durissima | PD | tree |
| Teclea_verdoorniana | PD | tree |
| Terminalia_ivorensis | PD | tree |
| Terminalia_superba | PD | tree |
| Tetrapleura_chevalieri | PD | tree |
| Tetrapleura_tetraptera | MD | tree |
| Tetrorchidium_didymostemon | MD | tree |
| Tieghemella_heckelii | MD | tree |
| Treculia_africana | PD | tree |
| Trichilia_heudelotti | PD | tree |
| Trichilia_lanata | PD | tree |
| Trichilia_prieureana | PD | tree |
| Trichoscypha_arborea | PD | tree |
| Trichoscypha_yapoensis | PD | tree |
| Trilepisium_angolensis | PD | tree |
| Triplochiton_cleroxylon | PD | tree |
| Turraeanthus_africana | PD | tree |
| Uapaca_esculenta | PD | tree |
| Uapaca_guineensis | PD | tree |
| Uapaca_heudelotii | PD | tree |
| Uapaca_paludosa | PD | tree |
| Vitex_fosteri | PD | tree |
| Vitex_micrantha | PD | tree |
| Voacanga_africana | PD | tree |
| Xylia_evansii | PD | tree |
| Xylopia_aethiopica | ND | tree |
| Xylopia_quintasii | ND | tree |
| Xylopia_rubescens | ND | tree |
| Xylopia_staudtii | ND | tree |
| Xylopia_villosum | ND | tree |
| Zanthoxylum_macrophylla | ND | tree |
| Zanthoxylum_microphylla | ND | tree |
| Zanthoxylum_rubescens | MPD | tree |
| Abrus_precatorius | MPD | tree |
| Adenanthera_pavonina | MPD | tree |
| Adinandra_dumosa | MPD | tree |
| Aglaia_ridleyi | MPD | tree |
| Alstonia_angustiloba | MPD | tree |
| Anisophyllea_disticha | MPD | tree |
| Antiaris_toxicaria | MPD | tree |
| Antidesma_cuspidatum | PD | tree |
| Ardisia_colorata | PD | tree |
| Ardisia_crenata | PD | tree |
| Artocarpus_altilis | PD | tree |
| Azadirachta_excelsa | PD | tree |
| Baccaurea_motleyana | PD | tree |
| Baccaurea_parviflora | PD | tree |
| Bhesa_paniculata | PD | tree |
| Bischofia_javanica | PD | tree |
| Caesalpinia_sappan | PD | tree |
| Callerya_atropurpurea | PD | tree |
| Calophyllum_inophyllum | PD | tree |
| Canarium_littorale | PD | tree |
| Canthium_glabrum | PD | tree |
| Careya_arborea | PD | tree |
| Clerodendrum_villosum | PD | tree |
| Cratoxylum_cochinchinense | ND | tree |
| Dimocarpus_longan | PD | tree |
| Diospyros_confertiflora | PD | tree |
| Dipterocarpus_cornutus | PD | tree |
| Dipterocarpus_grandiflorus | PD | tree |
| Dipterocarpus_kunstleri | PD | tree |
| Dracontomelum_mangiferum | ND | tree |
| Dryobalanops_aromatica | PD | tree |
| Durio_zibethinus | PD | tree |
| Dysoxylum_cauliflorum | PD | tree |
| Elaeocarpus_stipularis | PD | tree |
| Erythrina_variegata | PD | tree |
| Eugenia_grandis | MPD | tree |
| Eugenia_jambos | MPD | tree |
| Eugenia_operculata | PD | tree |
| Ficus_benjamina | PD | tree |
| Ficus_chartacea | PD | tree |
| Ficus_grossulariodes | PY | tree |
| Ficus_microcarpa | PY | tree |
| Ficus_virens | PY | tree |
| Garcinia_griffithii | ND | tree |
| Garcinia_mangostana | ND | tree |
| Gironniera_parviflora | PD | tree |
| Gnetum_gnemon | PD | tree |
| Gomphia_serrata | PD | tree |
| Gymnacranthera_eugeniifolia | PD | tree |
| Gyrocarpus_americanus | ND | tree |
| Hopea_ferrea | PD | tree |
| Hopea_odorata | ND | tree |
| Intsia_palembanica | ND | tree |
| Knema_laurina | ND | tree |
| Lagerstroemia_speciosa | ND | tree |
| Leea_indica | ND | tree |
| Leptospermum_flavescens | ND | tree |
| Lithocarpus_ewyckii | PD | tree |
| Lithocarpus_lucidus | PD | tree |
| Macaranga_tanarius | PD | tree |
| Macaranga_triloba | ND | tree |
| Magnolia_elegans | PD | tree |
| Mesua_ferrea | PD | tree |
| Microdesmis_caseariifolia | PD | tree |
| Micromelum_minutum | PD | tree |
| Mimusops_elengi | PD | tree |
| Murraya_paniculata | PD | tree |
| Nephelium_lappaceum | ND | tree |
| Nephelium_malaiense | PY | tree |
| Nothaphoebe_umbelliflora | ND | tree |
| Ochanostachys_amentacea | PD | tree |
| Oroxylum_indicum | PD | tree |
| Palaquium_gutta | PD | tree |
| Parkia_javanica | PD | tree |
| Parkia_speciosa | PD | tree |
| Pellacalyx_saccardianus | PD | tree |
| Peltophorum_pterocarpum | PD | tree |
| Pericopsis_mooniana | PD | tree |
| Phaeanthus_ophthalmicus | PD | tree |
| Phyllanthus_emblica | PD | tree |
| Pithecellobium_clypearia | PD | tree |
| Pittosporum_ferrugineum | PD | tree |
| Podocarpus_imbricatus | PD | tree |
| Podocarpus_neriifolius | ND | tree |
| Pometia_pinnata | PY | tree |
| Pongamia_pinnata | ND | tree |
| Porterandia_anisophylla | PD | tree |
| Pouteria_maingayi | ND | tree |
| Pterocarpus_indicus | ND | tree |
| Rhodamnia_cinerea | PD | tree |
| Rhodomyrtus_tomentosa | PD | tree |
| Sandoricum_koetjape | ND | tree |
| Sapium_indicum | ND | tree |
| Scaphium_macropodum | PD | tree |
| Schima_wallichii | PD | tree |
| Shorea_argentifolia | PD | tree |
| Shorea_assamica | PD | tree |
| Shorea_leprosula | PD | tree |
| Shorea_macrophylla | PD | tree |
| Shorea_macroptera | PD | tree |
| Shorea_ovalis | ND | tree |
| Shorea_palembanica | ND | tree |
| Shorea_parvifolia | PD | tree |
| Shorea_roxburghii | PD | tree |
| Shorea_siamensis | PD | tree |
| Sophora_tomentosa | PD | tree |
| Sterculia_coccinea | ND | tree |
| Sterculia_foetida | PD | tree |
| Terminalia_belirica | PD | tree |
| Terminalia_calamansanai | PD | tree |
| Xylocarpus_granatum | PD | tree |
| Acalypha_macrostachya | PD | tree |
| Adelia_triloba | ND | tree |
| Aegiphila_panamensis | PY | tree |
| Alibertia_edulis | PY | tree |
| Ardisia_bartlettii | MPD | tree |
| Bertiera_guianensis | MPD | tree |
| Brunfelsia_chocoensis | PY | tree |
| Calycolpus_warscewiczianus | PY | tree |
| Casearia_arguta | PY | tree |
| Casearia_guianensis | PD | tree |
| Cassia_fruticosa | ND | tree |
| Cassipourea_elliptica | ND | tree |
| Cestrum_latifolium | PD | tree |
| Cestrum_megalophyllum | PD | tree |
| Coccoloba_acuminata | PD | tree |
| Cordia_lasiocalyx | ND | tree |
| Cordia_panamensis | ND | tree |
| Cordia_spinescens | PD | tree |
| Cyphomandra_hartwegii | ND | tree |
| Dalbergia_brownei | PD | tree |
| Erythroxylum_multiflorum | PD | tree |
| Erythroxylum_panamense | PD | tree |
| Eugenia_venezuelensis | PY | tree |
| Hamelia_patens | PY | tree |
| Heisteria_acuminata | PY | tree |
| Helicteres_guazumaefolia | PY | tree |
| Herrania_purpurea | PY | tree |
| Hybanthus_prunifolius | PY | tree |
| Miconia_affinis | PY | tree |
| Miconia_impetiolaris | PY | tree |
| Miconia_lonchophylla | ND | tree |
| Miconia_prasina | ND | tree |
| Muntingia_calabura | PY | tree |
| Ouratea_lucens | PY | tree |
| Palicourea_guianensis | PY | tree |
| Palicourea_triphylla | PY | tree |
| Picramnia_latifolia | PY | tree |
| Piper_aduncum | ND | tree |
| Piper_tuberculatum | MPD | tree |
| Posoqueria_latifolia | ND | tree |
| Psidium_guajava | PY | tree |
| Psychotria_brachiata | ND | tree |
| Psychotria_grandis | PD | tree |
| Quassia_amara | ND | tree |
| Randia_formosa | ND | tree |
| Rinorea_squamata | MPD | tree |
| Rinorea_sylvatica | ND | tree |
| Siparuna_guianensis | ND | tree |
| Siparuna_pauciflora | MPD | tree |
| Solanum_asperum | PD | tree |
| Solanum_hayesii | PD | tree |
| Solanum_ochraceo-ferrugineum | PY | tree |
| Stemmadenia_grandiflora | PY | tree |
| Stylogyne_standleyi | ND | tree |
| Talisia_nervosa | ND | tree |
| Thevetia_ahouai | ND | tree |
| Tovomitopsis_nicaraguensis | PY | tree |
| Triumfetta_bogotensis | PY | tree |
| Vernonia_patens | PY | tree |
| Vismia_billbergiana | ND | tree |
| Albizia_guachapele | ND | tree |
| Alchornea_costaricensis | PY | tree |
| Alchornea_latifolia | ND | tree |
| Allophylus_psilospermus | ND | tree |
| Alseis_blackiana | ND | tree |
| Anacardium_excelsum | PD | tree |
| Anacardium_occidentale | PD | tree |
| Andira_inermis | PY | tree |
| Annona_acuminata | ND | tree |
| Annona_glabra | ND | tree |
| Annona_hayesii | ND | tree |
| Annona_muricata | ND | tree |
| Annona_purpurea | ND | tree |
| Antirhea_trichantha | PY | tree |
| Apeiba_membranacea | ND | tree |
| Apeiba_tibourbou | ND | tree |
| Aspidosperma_cruenta | ND | tree |
| Astrocaryum_standleyanum | PD | tree |
| Astronium_graveolens | PD | tree |
| Bactris_barronis | ND | tree |
| Bactris_coloradonis | PY | tree |
| Beilschmiedia_pendula | PD | tree |
| Bombacopsis_sessilis | ND | tree |
| Bunchosia_cornifolia | ND | tree |
| Callichlamys_latifolia | ND | tree |
| Calopogonium_mucunioides | MPD | tree |
| Carica_cauliflora | MPD | tree |
| Carica_papaya | ND | tree |
| Casearia_aculeata | MPD | tree |
| Casearia_arborea | MPD | tree |
| Cavanillesia_platanifolia | PY | tree |
| Cecropia_insignis | PY | tree |
| Cecropia_longipes | PD | tree |
| Cecropia_obtusifolia | ND | tree |
| Cecropia_peltata | ND | tree |
| Cespedesia_macrophylla | PD | tree |
| Chamaedorea_allenii | ND | tree |
| Chamaedorea_wendlandiana | PY | tree |
| Chrysophyllum_cainito | PY | tree |
| Chrysophyllum_panamense | PD | tree |
| Coccoloba_manzanillensis | ND | tree |
| Cochlospermum_vitifolium | PY | tree |
| Cordia_alliodora | PY | tree |
| Cordia_bicolor | ND | tree |
| Couratari_panamensis | ND | tree |
| Crossopetalum_eucymosa | PD | tree |
| Cupania_rufescens | PY | tree |
| Cupania_sylvatica | ND | tree |
| Dalbergia_retusa | MPD | tree |
| Dendropanax_arboreus | ND | tree |
| Desmopsis_panamensis | ND | tree |
| Didymopanax_morototoni | MPD | tree |
| Diospyros_artanthifolia | PY | tree |
| Dipteryx_panamensis | PY | tree |
| Drypetes_standleyi | PD | tree |
| Enterolobium_cyclocarpum | ND | tree |
| Enterolobium_schomburgkii | PD | tree |
| Erythrina_berteroana | ND | tree |
| Erythrina_fusca | PY | tree |
| Eugenia_coloradensis | PD | tree |
| Eugenia_nesiotica | PD | tree |
| Eugenia_oerstedeana | PD | tree |
| Ficus_dugandii | PD | tree |
| Ficus_glabrata | PD | tree |
| Ficus_insipida | PD | tree |
| Ficus_maxima | PD | tree |
| Ficus_yoponensis | PD | tree |
| Fissicalyx_fendleri | PD | tree |
| Guapira_standleyanum | PD | tree |
| Guarea_grandifolia | PD | tree |
| Guatteria_amplifolia | ND | tree |
| Gustavia_superba | ND | tree |
| Hampea_appendiculata | ND | tree |
| Hasseltia_floribunda | PD | tree |
| Heisteria_concinna | PD | tree |
| Henriettea_succosa | ND | tree |
| Henriettella_fasicularis | ND | tree |
| Henriettella_sylvestris | ND | tree |
| Hirtella_americana | ND | tree |
| Hirtella_triandra | ND | tree |
| Hura_crepitans | ND | tree |
| Hyeronima_laxiflora | ND | tree |
| Hymenaea_courbaril | ND | tree |
| Inga_fagifolia | ND | tree |
| Inga_goldmanii | PD | tree |
| Inga_minutula | PD | tree |
| Inga_multijuga | PD | tree |
| Inga_pauciflora | PD | tree |
| Inga_quaternata | PD | tree |
| Inga_sapindoides | PD | tree |
| Inga_spectabilis | PD | tree |
| Inga_umbellifera | PD | tree |
| Lacistema_aggregatum | PD | tree |
| Lacmellea_panamensis | PD | tree |
| Laetia_procera | PD | tree |
| Laetia_thamnia | PD | tree |
| Lafoensia_punicifolia | PD | tree |
| Licania_platypus | PD | tree |
| Lindackeria_laurina | PD | tree |
| Lonchocarpus_pentaphyllus | PD | tree |
| Lonchocarpus_velutinus | PD | tree |
| Luehea_seemannii | PD | tree |
| Lycianthes_maxonii | PD | tree |
| Maripa_panamensis | PD | tree |
| Maytenus_schippii | ND | tree |
| Myrcia_gatunensis | ND | tree |
| Nectandra_gentlei | PD | tree |
| Ocotea_skutchii | PD | tree |
| Ocotea_oblonga | PD | tree |
| Ocotea_pyramidata | PD | tree |
| Oenocarpus_panamanus | ND | tree |
| Olmedia_aspera | ND | tree |
| Ormosia_coccinea | ND | tree |
| Ormosia_macrocalyx | PY | tree |
| Perebea_xanthochyma | PY | tree |
| Persea_americana | PY | tree |
| Pithecellobium_macradenium | PY | tree |
| Pithecellobium_mangense | PY | tree |
| Pithecellobium_rufescens | PY | tree |
| Platypodium_elegans | PY | tree |
| Pourouma_guianensis | PY | tree |
| Pouteria_sapota | PY | tree |
| Pouteria_unilocularis | PY | tree |
| Prioria_copaifera | PY | tree |
| Protium_panamense | PY | tree |
| Protium_tenuifolium | PY | tree |
| Pseudobombax_septenatum | PY | tree |
| Psidium_friedrichsthalianum | PY | tree |
| Pterocarpus_officinalis | PY | tree |
| Quararibea_asterolepis | PD | tree |
| Quararibea_pterocalyx | PD | tree |
| Randia_armata | PD | tree |
| Rheedia_acuminata | PD | tree |
| Rheedia_edulis | PD | tree |
| Roupala_montana | PD | tree |
| Saurauia_laevigata | PD | tree |
| Sloanea_terniflora | PD | tree |
| Sorocea_affinis | PD | tree |
| Spondias_radlkoferi | PD | tree |
| Sterculia_apetala | PD | tree |
| Swartzia_simplex | PD | tree |
| Synechanthus_warscewiczianus | PD | tree |
| Tabebuia_guayacan | PD | tree |
| Tabebuia_rosea | PD | tree |
| Tabernaemontana_arborea | PD | tree |
| Tachigali_versicolor | PD | tree |
| Talisia_princeps | PD | tree |
| Terminalia_amazonica | PD | tree |
| Terminalia_chiriquensis | PD | tree |
| Ternstroemia_tepezapote | PD | tree |
| Tetragastris_panamensis | PD | tree |
| Tetrathylacium_johansenii | MD | tree |
| Tocoyena_pittieri | MD | tree |
| Tovomita_longifolia | MD | tree |
| Trattinnickia_aspera | PY | tree |
| Trichilia_tuberculata | PY | tree |
| Trichospermum_galeottii | PY | tree |
| Triplaris_cumingiana | PD | tree |
| Unonopsis_panamensis | PD | tree |
| Unonopsis_pittieri | PD | tree |
| Virola_sebifera | PD | tree |
| Virola_surinamensis | PD | tree |
| Vismia_macrophylla | PD | tree |
| Xylopia_macrantha | PD | tree |
| Xylosma_oligandrum | PD | tree |
| Zanthoxylum_belizense | PD | tree |
| Zuelania_guidonia | PD | tree |
| Cynodon dactylon | ND | herb |
| Cercocarpus montanus | PD | tree |
| Larix decidua | PD | tree |
| pogogyne abramsii | PY | herb |
| Hordeum vulgare | PD | herb |
| *Onobrychis viciifolia* | PY | herb |
| Panicum virgatum L | PD | herb |
| *Aegilops neglecta* | PD | herb |
| *Aegilops geniculata* | PD | herb |
| *Aegilops riuncialis* | PD | herb |
| *Brassica rapa* | PD | herb |
| *Crotalaria pumila* | PY | Shrub |
| *Medicago sativa* | PY | herb |
| *Avena sativa* | PD | herb |
| *Spartina densiflora* | PD | Shrub |
| *Atriplex portulacoides* | PD | Shrub |
| Castanea sativa | PD | tree |
| Triticum aestivum | PD | herb |
| *Castanea sativa* | PD | tree |
| *Gmelina Arborea* | ND | tree |
| Solanum tuberosum | PD | herb |
| Vicia angustifolia | PY | herb |
| Afzelia quanzensis | PD | tree |
| Allium cepa | ND | herb |
| *Piper peltatum* | MD | shrub |
| *Miconia argentea* | ND |  |
| *Piper dilatatum* | MD | shrub |
| *Alseis blackiana* | ND | tree |
| *Cecropia peltata* | ND | tree |
| *Cecropia obtusifolia* | PY | tree |
| *Cecropia insignis* | ND | tree |
| *Luehea seemannii* | PY | tree |
| *Solanum hayesii* | PD | shrub |
| *Trichospermum mexicanum* | PY | tree |
| *Trema micrantha* | PD | tree |
| *Guazuma ulmifolia* | PY | tree |
| *Ochroma pyramidale* | PY | tree |
| *Apeiba membranacea* | PD | tree |
| *Pseudobombax septenatum* | ND | tree |
| Potamogeton pectinatus | PD |  |
| Sarracenia purpurea ssp. Purpurea | ND | shrub |
| Sarracenia ssp. Venosa | MPD | shrub |
| Sarracenia fiava | MPD | shrub |
| Sarracenia minor | MPD | shrub |
| Sarracenia rubra ssp. Rubra | MPD | shrub |
| Sarracenia jonesii | MPD | shrub |
| Sarracenia leucophylla | MPD | shrub |
| Sarracenia psittacina | MPD | shrub |
| Sarracenia alata | MPD | shrub |
| Cistus salviifolius | PY | shrub |
| Virola koschnyi | MPD | tree |
| Rice | PD | herb |
| Arachis hypogaea | PD | herb |
| Glycine max | PD | herb |
| Vicia cracca | PY | shrub |
| Spinacia oleracea | PD | herb |
| *Lens culinaris* Medik. cvs. Jor-1 | PD | herb |
| *Lens culinaris* Medik. cvs. Jor-2 | PD | herb |
| *Lens culinaris* Medik. cvs. Jor-3 | PD | herb |
| Lens culinaris Medik. cvs. Jor-4 | PD | herb |
| *Acacia falciformis* | PY | tree |
| *Acanthocephala terminalis* | PY | tree |
| *Goodia lotifolia* | PY | tree |
| *Acacia venulosa* | PY | tree |
| *Acacia ulicifolia* | PY | tree |
| *Acacia viscidula* | PY | tree |
| *Gompholobium latifolium* | PY | shrub |
| *Dillwynia phylicoides* | PY | shrub |
| Vaccinium angustifolium | MPD | shrub |
| radish | PY | herb |
| cercis canadensis | PY | tree |
| Zea mays | PD | herb |
| Sorghum bicolar | PD | herb |
| soybean | PD | herb |
| Picea glauca | PD | tree |
| Atriplex cordobensis | PD | shrub |
| Bossiaea aquifolium | PY |  |
| Chorizema ilicifolium | PY |  |
| Daviesia horrida | PY |  |
| Gastrolobium spinosum | PY |  |
| Gompholobium knightianum | PY |  |
| Hovea chorizemifolia | PY |  |
| Kennedia coccinea | PY |  |
| Sphaerolobium vimineum | PY |  |
| *Rhamnus davurica* | PY | herb |
| *Setaria viridis* | PD | herb |
| dipterocarpus macrocarpus | ND | tree |
| TRAGOPOGON PRATENSIS | PD | herb |
| rumex crispus | PD | herb |
| rumex obtusifolius | PD | herb |
| Cannabis sativa | PD | herb |
| Cordia africana | PD | tree |
| Calluna vulgaris | PY | shrub |
| Campanula rotundifolia | MD | shrub |
| Carlina vulgaris | PD | shrub |
| Danthonia decumbens | PD | shrub |
| Dianthus deltoides | PD | shrub |
| Hieracium pilosella | PD | shrub |
| Lotus corniculatus | PY | shrub |
| Plantago media | PD | shrub |
| Polygala vulgaris | PD | shrub |
| Thymus serpyllum | PD | shrub |
| Hyptis suaveolens | PD | shrub |
| actaea rubra(Ait.) Willd | ND | shrub |
| Sorbus scopulina Greene | PD | shrub |
| Symphoricarpos albus (L.) Blake | MPD | shrub |
| Disporum trachycarpum | MPD | shrub |
| Smilacina racemosa | MPD | shrub |
| Smilacina stellata | MPD | shrub |
| Streptopus amplexifolius | MPD | shrub |
| Elaeagnus umbellata | PD | tree |
| Hydrangea paniculata | PD | tree |
| Lotus corniculstus | PY |  |
| *Vigna radiata* | PY | shrub |
| *Vigna mungo* | PY | shrub |
| Brassica juncea | PD | shrub |
| *Lagochilus ilicifolium* | PY | shrub |
| *Allium ramosum* | PD | shrub |
| *Allium polyrhizum* | PD | shrub |
| *Linum stelleroides* | PD | shrub |
| *Stipa breviflora* | PD | shrub |
| *Allium tenuissimum* | PD | shrub |
| *Haplophyllum dauricum* | PD | shrub |
| *Kochia prastrata* | PD | shrub |
| *Bassia dasyphylla* | PD | shrub |
| *Amaranthus retroflexus* | PD | shrub |
| *Heteropappus altaicus* | PD | shrub |
| *Potentilla tanacetifolia* | PD | shrub |
| *Plantago depressa* | PD | shrub |
| *Artemisia sieversiana* | ND | shrub |
| *Neopallasia pectinata* | PD | shrub |
| *Potentilla multicaulis* | PD | shrub |
| *Artemisia frigida* | PD | shrub |
| *Artemisia mongolica* | PD | shrub |
| *Artemisia annua* | PD | shrub |
| *Artemisia scoparia* | PD | shrub |
| Ceratoides lanata | PD | shrub |
| Artemisia cana | PD | shrub |
| *Escontria chiotilla* | PD | shrub |
| Nicotiana tabacum | PD | shrub |
| Cannabis *sativa* | PD | shrub |
| vaccinium corymbosum | PD | shrub |
| Populus deltoid | ND | tree |
| Potamogeton pectinatus | PD | shrub |
| Potamogeton pectinatus | ND | shrub |
| Peucedanum oreoselinum | MPD | shrub |
| Leymus arenarius | PD | shrub |
| Artemisia sieversiana Willd | ND | shrub |
| Cremanthodium pleurocaule | PD | shrub |
| Ligularia przewalskii | PD | shrub |
| Senecio diversipinnus Ling | PD | shrub |
| Saussurea hieracioides Hook | PD | shrub |
| Saussurea iodostegia Hance | PD | shrub |
| Saussurea japonica | ND | shrub |
| Saussurea morifolia Chen | PD | shrub |
| Saussurea mongolica(Franch.) | PD | shrub |
| Saussurea parviflora (Poir.) DC | PD | shrub |
| Saussurea variiloba Ling. | PD | shrub |
| Tripolium vulgare Ness. | PD | shrub |
| Ranunculus brotherusii var. tanguticus | MPD | shrub |
| Trollius farreri Stapf | MPD | shrub |
| Dracocephalum ruyschiana | PD | shrub |
| Laniophlomis rotate (Benth.) | PD | shrub |
| Salvia przewalskii Maxim | PD | shrub |
| Hedysarum tanguticum Fedtsch | PY | shrub |
| Medicago minima Bart | PY | shrub |
| Vicia angustifolia L. | PY | shrub |
| Agrostis alba | ND | shrub |
| Agrostis hugouiana | PD | shrub |
| Agrostis stolonifera | PD | shrub |
| Poa poophagorum | PD | shrub |
| Poa botryoides | PD | shrub |
| Poa schoenites | PD | shrub |
| Helictotrichon schellianum | PD | shrub |
| Deyeuxia arundinacea | ND | shrub |
| Ptilagrostis dichotoma var. roshevitsiana | PY | shrub |
| Koeleria cristata | ND | shrub |
| Deschampsia caespitosa | PD | shrub |
| Poa declinata | PD | shrub |
| Poa pratensis | PD | shrub |
| Agrostis trinii | PD | shrub |
| Helictotrichon leianthum | PD | shrub |
| Mellica scabrosa | PD | shrub |
| Ptilagrostis junatovii | PD | shrub |
| Festuca ovina | PD | shrub |
| Orinus kokonorica | PD | shrub |
| Deyeuxia scabrescens | PD | shrub |
| Festuca rubra | PD | shrub |
| Poa attenuate var. vivipara | PD | shrub |
| Festuca sinensis | PD | shrub |
| Achnatherum splendens | ND | shrub |
| Ptilagrostis dichotoma | PY | shrub |
| Achnatherum inebrians | ND | shrub |
| Ptilagrostis mongholica | PD | shrub |
| Stipa purpurea | ND | shrub |
| Stipa aliena | ND | shrub |
| Helictotrichon tibeticum | PD | shrub |
| Oryzopsis munroi | PD | shrub |
| Achnatherum sibiricum | ND | shrub |
| Elymus dahuricus | ND | shrub |
| Bromus magnus | PD | shrub |
| Bromus tectorum | PD | shrub |
| Roegneria stricta | PD | shrub |
| Brachypodium sylvaticum var. gracile | PD | shrub |
| Roegneria kokonorica | PD | shrub |
| Brachypodium sylvaticum | PD | shrub |
| Bromus sinensis | PD | shrub |
| Stipa capillacea | ND | shrub |
| Stipa przewalskyi | ND | shrub |
| Aneurolepidium dasystachys | PD | shrub |
| Elymus tangutorum | PD | shrub |
| Roegneria nutans | PD | shrub |
| Eupatorium buniifolium | PD | herb |
| Parthenium hysterophorus | PD | herb |
| Zinnia peruviana | PD | herb |
| Flourensia campestris | PD | shrub |
| Flourensia oolepis | PD | shrub |
| Xanthorrhoea johnsonii | PD | tree |
| Cercocarpus betuloides | PD | Herb |
| Raphanus sativus | PD | Herb |
| Nicotiana tabacum | PD | Herb |
| Andropogon hallii | PD | Herb |
| Bouteloua curtipendula | PD | Herb |
| Bouteloua gracilis | PD | Herb |
| Panicum virgatum | PD | Herb |
| Sorghastrum nutans | PD | Herb |
| *Halimione portulacoides* | PD | Shrub |
| ceiba aesculifolia | PD | tree |
| Zostera marina | PD | herb |
| ANABASIS APHYLLA | PD | shrub |
| Terminalia bellerica | PD | tree |
| Betula lenta | PD | tree |
| Liquidambar styraciflua | PD | tree |
| Acer palmatum | PD | tree |
| Ailanthus altissima | PD | tree |
| Liriodendron tulipifera | PD | tree |
| Acer saccharum | PD | tree |
| Acer pseudoplatanus | PD | tree |
| Acer platanoides | PD | tree |
| Quercus velutina | PD | tree |
| Quercus alba | PD | tree |
| Quercus rubra | PD | tree |
| *Actaea spicata* | PD | herb |
| Solidago canadensi | PD | herb |
| Hieraciumpratens | PD | herb |
| Hypericum perforatum | PD | herb |
| Poa pratensi | PD | herb |
| mentha arvensis | PD | herb |
| centaurea nigra | PD | herb |
| echium vulgare | PD | herb |
| Tragopogon dubius | PD | herb |
| Lolium perenne | PD | herb |
| *Sorghum bicolar* | PD | herb |
| cicer milkvetch | PD | herb |
| cicer milkvetc | PD | herb |
| falcatus milkvetch | PD | herb |
| *Pterocarpus marsupium* | PD | tree |
| Pinus strobus | PD | tree |
| P. faxlucens | PD | tree |
| P. flava | PD | tree |
| P. papantlensis | PD | tree |
| P. limonensis | PD | tree |
| Psathyrosttchys junce | PD | herb |
| *Salsola collina* | PD | shrub |
| *Axyris amaranthoides* | PD | herb |
| *Chenopodium foetidum* | PD | herb |
| *Allium przewalskianum* | PD | herb |
| *Allium cyaneum* | PD | herb |
| *Ligusticum moniliforme* | PD | herb |
| *Senecio dubitabilis* | PD | herb |
| *Arctium lappa* | PD | herb |
| *Heteropappus altaicus* | PD | herb |
| *Heteropappus gouldii* | PD | herb |
| *Artemisia desertorum* | PD | herb |
| *Picris hieracioides* | PD | herb |
| *Aster souliei* | PD | herb |
| *Leontopodium leontopodioides* | PD | herb |
| *Artemisia phaeolepis* | PD | herb |
| *Sonchus transcaspicus* | PD | herb |
| *Leontopodium calocephalum* | PD | herb |
| *Sinacalia tangutica* | PD | herb |
| *Senecio argunensis* | PD | herb |
| *Artemisia argyi* | PD | herb |
| *Cirsium setosum* | PD | herb |
| *Heteropappus crenatifolius* | PD | herb |
| *Ligularia sagitta* | PD | herb |
| *Leontopodium souliei* | PD | herb |
| *Artemisia scoparia* | PD | herb |
| *Saussurea parviflora* | PD | herb |
| *Prenanthes tatarinowii* | PD | herb |
| *Ligularia przewalskii* | PD | herb |
| *Artemisia mongolica* | PD | herb |
| *Ligularia virgaurea* | PD | herb |
| *Saussurea stella* | PD | herb |
| *Cynoglossum amabile* | PD | herb |
| *Lepidium apetalum* | PD | herb |
| *Descurainia sophia* | PD | herb |
| *Torularia humilis* | PD | herb |
| *Thlaspi arvense* | PD | herb |
| *Draba eriopoda* | PD | herb |
| *Dipsacus japonicus* | PD | herb |
| *Silene pterosperma* | PD | herb |
| *Cerastium fontanum* | PD | herb |
| *Stellaria media* | PD | herb |
| *Silene aprica* | PD | herb |
| *Stellaria graminea* | PD | herb |
| *Parnassia brevistyla* | PD | herb |
| *Rhodiola kirilowii* | PD | herb |
| *Orostachys fimbriatus* | PD | herb |
| *Carex chlorostachys* | PD | herb |
| *Carex enervis* | PD | herb |
| *Blysmus sinocompressus* | PD | herb |
| *Hypericum przewalskii* | PD | herb |
| *Nepeta prattii* | PD | herb |
| *Dracocephalum tanguticum* | PD | herb |
| *Elsholtzia densa* | PD | herb |
| *Salvia roborowskii* | PD | herb |
| *Galeopsis bifida* | PD | herb |
| *Lilium pumilum* | PD | herb |
| *Linum perenne* | PD | herb |
| *Epilobium palustre* | PD | herb |
| *Epilobium angustifolium* | PD | herb |
| *Pedicularis alaschanica* | PD | herb |
| *Pedicularis szetschuanica* | PD | herb |
| *Euphrasia* sp. | PD | herb |
| *Pedicularis chinensis* | PD | herb |
| *Pedicularis cristatella* | PD | herb |
| *Pedicularis polyodonta* | PD | herb |
| *Pedicularis cheilanthifolia* | PD | herb |
| *Pedicularis semitorta* | PD | herb |
| *Pedicularis brevilabris* | PD | herb |
| *Euphrasia regelii* | PD | herb |
| *Pedicularis longiflora* | PD | herb |
| *Pedicularis rudis* | PD | herb |
| *Pedicularis rhinanthoides* | PD | herb |
| *Plantago depressa* | PD | herb |
| *Veronica eriogyne* | PD | herb |
| *Veronica biloba* | PD | herb |
| *Scrofella chinensis* | PD | herb |
| *Plumbagella micrantha* | PD | herb |
| *Poa poophagorum* | PD | herb |
| *Elymus sibiricus* | PD | herb |
| *Roegneria breviglumis* | PD | herb |
| *Bromus magnus* | PD | herb |
| *Poa annua* | PD | herb |
| *Bromus tectorum* | PD | herb |
| *Festuca sinensis* | PD | herb |
| *Stipa baicalensis* | PD | herb |
| *Roegneria parvigluma* | PD | herb |
| *Festuca ovina* | PD | herb |
| *Oryzopsis munroi* | PD | herb |
| *Stipa capillacea* | PD | herb |
| *Roegneria stricta* | PD | herb |
| *Stipa aliena* | PD | herb |
| *Bromus sp* | PD | herb |
| *Deschampsia caespitosa* | PD | herb |
| *Poa pratensis* | PD | herb |
| *Agrostis hugoniana* | PD | herb |
| *Stipa przewalskyi* | PD | herb |
| *Beckmannia syzigachne* | PD | herb |
| *Stipa bungeana* | PD | herb |
| *Trikeraia pappiformis* | PD | herb |
| *Trisetum clarkei* | PD | herb |
| *Agrostis gigantea* | PD | herb |
| *Brachypodium sylvaticum* | PD | herb |
| *Agrostis micrantha* | PD | herb |
| *Deyeuxia scabrescens* | PD | herb |
| *Aristida triseta* | PD | herb |
| *Deyeuxia flavens* | PD | herb |
| *Leymus secalinus* | PD | herb |
| *Polemonium coeruleum* | PD | herb |
| *Fagopyrum tataricum* | PD | herb |
| *Polygonum macrophyllum* | PD | herb |
| *Pomatosace filicula* | PD | herb |
| *Androsace mariae* | PD | herb |
| *Androsace erecta* | PD | herb |
| *Clematis tangutica* | PD | herb |
| *Potentilla tanacetifolia* | PD | herb |
| *Geum aleppicum* | PD | herb |
| *Potentilla bifurca* | PD | herb |
| *Sanguisorba officinalis* | PD | herb |
| *Agrimonia pilosa* | PD | herb |
| *Potentilla saundersiana* | PD | herb |
| *Potentilla multifida* | PD | herb |
| *Fragaria orientalis* | PD | herb |
| *Sibiraea laevigata* | PD | shrub |
| *Anisodus tanguticus* | PD | shrub |
| *Urtica triangularis* | PD | herb |
| *Agropyron desertorum* | PD | herb |
| C. microphylla | PY | Tree |
| H.laeve | PY | shrub |
| medicago lupulina | PY | Herb |
| *Vicia cracca* | PY | Herb |
| *Vicia angustifolia* | PY | Herb |
| *Tibetia himalaica* | PY | herb |
| *Hedysarum polybotrys* | PY | herb |
| *Astragalus polycladus* | PY | herb |
| *Caragana erinacea* | PY | Shrub |
| *Thermopsis lanceolala* | PY | herb |
| *Astragalus adsurgens* | PY | herb |
| *Oxytropis ochrocephala* | PY | herb |
| *Astragalus membranaceus* | PY | herb |
| *Medicago lupulina* | PY | herb |
| *Oxytropis kansuensis* | PY | herb |
| *Vicia unijuga* | PY | herb |
| *Astragalus melilotoides* | PY | Shrub |
| *Medicago ruthenica* | PY | herb |
| *Astragalus floridus* | PY | herb |
| *Astragalus przewalskii* | PY | herb |
| *Swertia tetraptera* | PY | herb |
| *Biebersteinia heterostemon* | PY | herb |
| *Erodium stephanianum* | PY | herb |
| *Juncus bufonius* | PY | herb |
| *Malva verticillata* | PY | herb |
| *Onobrychls* | PY | herb |
| Camelina sativa | ND | herb |
| *E. blakelyi* | ND | tree |
| *E. camaldulensis* | ND | tree |
| *E. melanophloia* | ND | tree |
| *E. melliodora* | ND | tree |
| *E. pilligaensis* | ND | tree |
| *E. populnea* | ND | tree |
| Chrysanthemumleucanthemu | ND | herb |
| Nitraria sphaerocarpa | ND | shrub |
| *Artemisia sieversiana* | ND | herb |
| *Taraxacum mongolicum* | ND | herb |
| *Saussurea nigrescens* | ND | herb |
| *Carduus nutans* | ND | herb |
| *Anaphalis lactea* | ND | herb |
| *Gerbera anandria* | ND | herb |
| *Carpesium lipskyi* | ND | herb |
| *Erigeron acer* | ND | herb |
| *Anaphalis margaritacea* | ND | herb |
| *Stellaria dianthifolia* | ND | herb |
| *Elymus nutans* | ND | herb |
| *Roegneria varia* | ND | herb |
| *Elymus tangutorum* | ND | herb |
| *Rumex patientia* | ND | herb |
| *Rumex nepalensis* | ND | herb |
| *Urtica cannabina* | ND | herb |
| Daucus carot | MD | herb |
| *Chenopodium hybridum* | MD | herb |
| *Heracleum millefolium* | MPD | herb |
| *Carum buriaticum* | MPD | herb |
| *Torilis japonica* | MPD | herb |
| *Carum carvi* | MPD | herb |
| *Bupleurum smithii* | MPD | herb |
| *Pleurospermum franchetianum* | MPD | herb |
| *Anthriscus sylvestris* | MPD | herb |
| *Seseli squarrulosum* | MPD | herb |
| *Pleurospermum hookeri* | MPD | herb |
| *Notopterygium forbesii* | MPD | herb |
| *Notopterygium incisum* | MPD | herb |
| *Tongoloa elata* | MPD | herb |
| *Adenophora himalayana* | MPD | herb |
| *Adenophora liliifolioides* | MPD | herb |
| *Adenophora* sp. | MPD | herb |
| *Halenia elliptica* | MPD | herb |
| *Comastoma pedunculatum* | MPD | herb |
| *Gentiana straminea* | MPD | herb |
| *Gentianopsis paludosa* | MPD | herb |
| *Lomatogonium macranthum* | MPD | herb |
| *Lomatogonium carinthiacum* | MPD | herb |
| *Gentiana striata* | MPD | herb |
| *Gentiana dahurica* | MPD | herb |
| *Gentianopsis contorta* | MPD | herb |
| *Gentianopsis* sp. | MPD | herb |
| *Gentiana choanantha* | MPD | herb |
| *Gentiana abaensis* | MPD | herb |
| *Geranium sibiricum* | MPD | herb |
| *Iris lactea* | MPD | herb |
| *Anemone rivularis* | MPD | herb |
| *Delphinium kamaonense* | MD | herb |
| *Trollius* sp. | MPD | herb |
| *Thalictrum minus* | MPD | herb |
| *Aconitum gymnandrum* | MPD | herb |
| *Thalictrum przewalskii* | MPD | herb |
| *Trollius farreri* | MPD | herb |
| *Thalictrum alpinum* | MPD | herb |
| *Thalictrum uncatum* | MPD | herb |
| *Thalictrum baicalense* | MPD | herb |
| *Aconitum sungpanense* | MPD | herb |
| *Delphinium souliei* | MPD | herb |
| *Cimicifuga foetida* | MPD | herb |
| Lotus corniculatus | PD | herb |
| CARTHAMUS TINCTORIUS | PD | herb |
| Onobrychis viciifolia | PD | herb |
| Anisodus tanguticus | PD | herb |
| Astragalus melilotoides | PY | herb |
| Sphallerocarpus | ND | herb |

**Additional references**

Adams, D. C., & Collyer, M. L. (2018). Phylogenetic ANOVA: group-clade aggregation, biological challenges, and a refined permutation procedure. Evolution, 72(6), 1204-1215.

Adams, D. C., & Collyer, M. L. (2018). Multivariate phylogenetic comparative methods: evaluations, comparisons, and recommendations. Systematic biology, 67(1), 14-31.

Bhattacharya, R., Lin, L., Patrangenaru, V., Bhattacharya, R., Lin, L., & Patrangenaru, V. (2016). Markov Chain Monte Carlo (MCMC) Simulation and Bayes Theory. A Course in Mathematical Statistics and Large Sample Theory, 325-332.

Cooper, A., Vehtari, A., Forbes, C., Simpson, D., & Kennedy, L. (2024). Bayesian cross-validation by parallel Markov chain Monte Carlo. Statistics and Computing, 34(4), 1-15.

Carpenter, B., Gelman, A., Hoffman, M. D., Lee, D., Goodrich, B., Betancourt, M., ... & Riddell, A. (2017). Stan: A probabilistic programming language. Journal of statistical software, 76.

Collyer, M. L., & Adams, D. (2021). Phylogenetically aligned component analysis.

Freckleton, R. P., Harvey, P. H., & Pagel, M. (2002). Phylogenetic analysis and comparative data: a test and review of evidence. The American Naturalist, 160(6), 712-726.

Felsenstein, J. (1985). Phylogenies and the comparative method. The American Naturalist, 125(1), 1-15.

Fernández‐Pascual, E., Carta, A., Mondoni, A., Cavieres, L. A., Rosbakh, S., Venn, S., ... & Jiménez‐Alfaro, B. (2021). The seed germination spectrum of alpine plants: a global meta‐analysis. New phytologist, 229(6), 3573-3586.

Gilks, W. R., & Roberts, G. O. (1996). Strategies for improving MCMC. Markov chain Monte Carlo in practice, 6, 89-114.

Gelman, A., Carlin, J. B., Stern, H. S., & Rubin, D. B. (1995). Bayesian data analysis. Chapman and Hall/CRC.

Gelman, A., Jakulin, A., Pittau, M. G., & Su, Y. S. (2008). A weakly informative default prior distribution for logistic and other regression models.

Garamszegi, L. Z. (Ed.). (2014). Modern phylogenetic comparative methods and their application in evolutionary biology: concepts and practice. Springer.

Hosmer Jr, D. W., Lemeshow, S., & Sturdivant, R. X. (2013). Applied logistic regression. John Wiley & Sons.

Hadfield, J. D. (2010). MCMC methods for multi-response generalized linear mixed models: the MCMCglmm R package. Journal of statistical software, 33, 1-22.

Hadfield, J. D., & Nakagawa, S. (2010). General quantitative genetic methods for comparative biology: phylogenies, taxonomies and multi‐trait models for continuous and categorical characters. Journal of evolutionary biology, 23(3), 494-508.

Ives, A. R., & Garland Jr, T. (2010). Phylogenetic logistic regression for binary dependent variables. Systematic biology, 59(1), 9-26.

Jombart, T., Devillard, S., & Balloux, F. (2010). Discriminant analysis of principal components: a new method for the analysis of genetically structured populations. BMC genetics, 11, 1-15.

Jolliffe, I. T., & Cadima, J. (2016). Principal component analysis: a review and recent developments. Philosophical transactions of the royal society A: Mathematical, Physical and Engineering Sciences, 374(2065), 20150202.

Paradis, E., Claude, J., & Strimmer, K. (2004). APE: analyses of phylogenetics and evolution in R language. Bioinformatics, 20(2), 289-290.

Pagel, M. (1999). Inferring the historical patterns of biological evolution. Nature, 401(6756), 877-884.

Pinheiro, J. (2011). nlme: Linear and nonlinear mixed effects models. R package version, 3, 1.

Rosbakh, S., Carta, A., Fernández‐Pascual, E., Phartyal, S. S., Dayrell, R. L., Mattana, E., ... & Baskin, C. (2023). Global seed dormancy patterns are driven by macroclimate but not fire regime. New Phytologist, 240(2), 555-564.

Revell, L. J. (2009). Size-correction and principal components for interspecific comparative studies. Evolution, 63(12), 3258-3268.

Revell, L. J. (2010). Phylogenetic signal and linear regression on species data. Methods in Ecology and Evolution, 1(4), 319-329.

Ringnér, M. (2008). What is principal component analysis?. Nature biotechnology, 26(3), 303-304.

Speagle, J. S. (2019). A conceptual introduction to Markov chain Monte Carlo methods. arXiv preprint arXiv:1909.12313.

Van Ravenzwaaij, D., Cassey, P., & Brown, S. D. (2018). A simple introduction to Markov Chain Monte–Carlo sampling. Psychonomic bulletin & review, 25(1), 143-154.

Wold, S., Esbensen, K., & Geladi, P. (1987). Principal component analysis. Chemometrics and intelligent laboratory systems, 2(1-3), 37-52.
